# Isotopic Consequences of Host–Guest Interactions; Noncovalent Chlorine Isotope Effects

**DOI:** 10.1021/acs.jpcb.0c10691

**Published:** 2021-02-11

**Authors:** Agata Paneth, Piotr Paneth

**Affiliations:** † Department of Organic Chemistry, Faculty of Pharmacy, Medical University of Lublin, Chodźki 4a, 20-093 Lublin, Poland; ‡ Institute of Applied Radiation Chemistry, Faculty of Chemistry, Lodz University of Technology, Żeromskiego 116, 90-924 Lodz, Poland

## Abstract

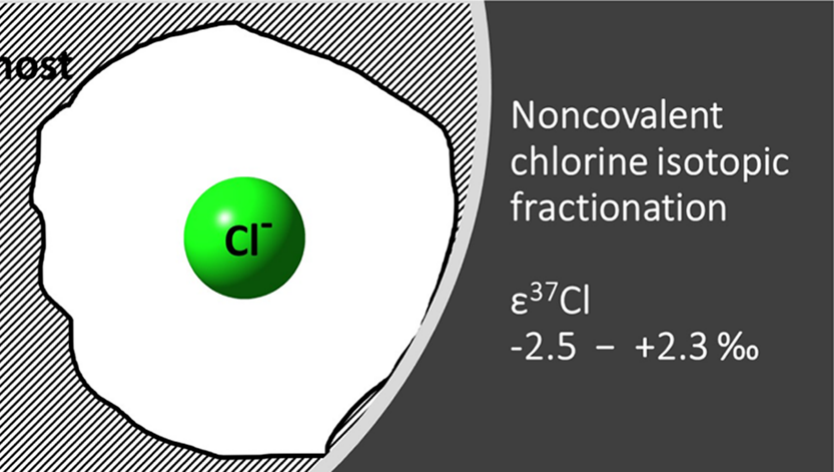

Although weak intermolecular interactions are the essence of most processes of key importance in medicine, industry, environment, and life cycles, their characterization is still not sufficient. Enzymatic dehalogenations that involve chloride anion interaction within a host–guest framework is one of the many examples. Recently published experimental results on host–guest systems provided us with models suitable to assess isotopic consequences of these noncovalent interactions. Herein, we report the influence of environmental and structural variations on chlorine isotope effects. We show that these effects, although small, may obscure mechanistic interpretations, as well as analytical protocols of dehalogenation processes.

## Introduction

Weak intermolecular interactions are the essence of many processes of key importance to life (e.g., the formation of Michaelis complexes during enzymatic reactions), industry (e.g., interactions within cavities, perovskites, or metal–organic frameworks (MOFs)), and medicine (e.g., host–guest interactions in drug delivery systems) to name a few most typical examples. It is thus not surprising that they are a continuous subject of vigorous studies. Due to our research involvement in enzymatic and environmentally oriented dehalogenation processes,^1−3^ particularly in chlorine isotopic fractionations, as well as the key role of chloride in many life-controlling processes,^4^ host–guest interactions between the chloride anion as a guest in different host frameworks are of special interest. In this respect, several recently published results provide a unique opportunity for their theoretical modeling.

**Figure 1:**
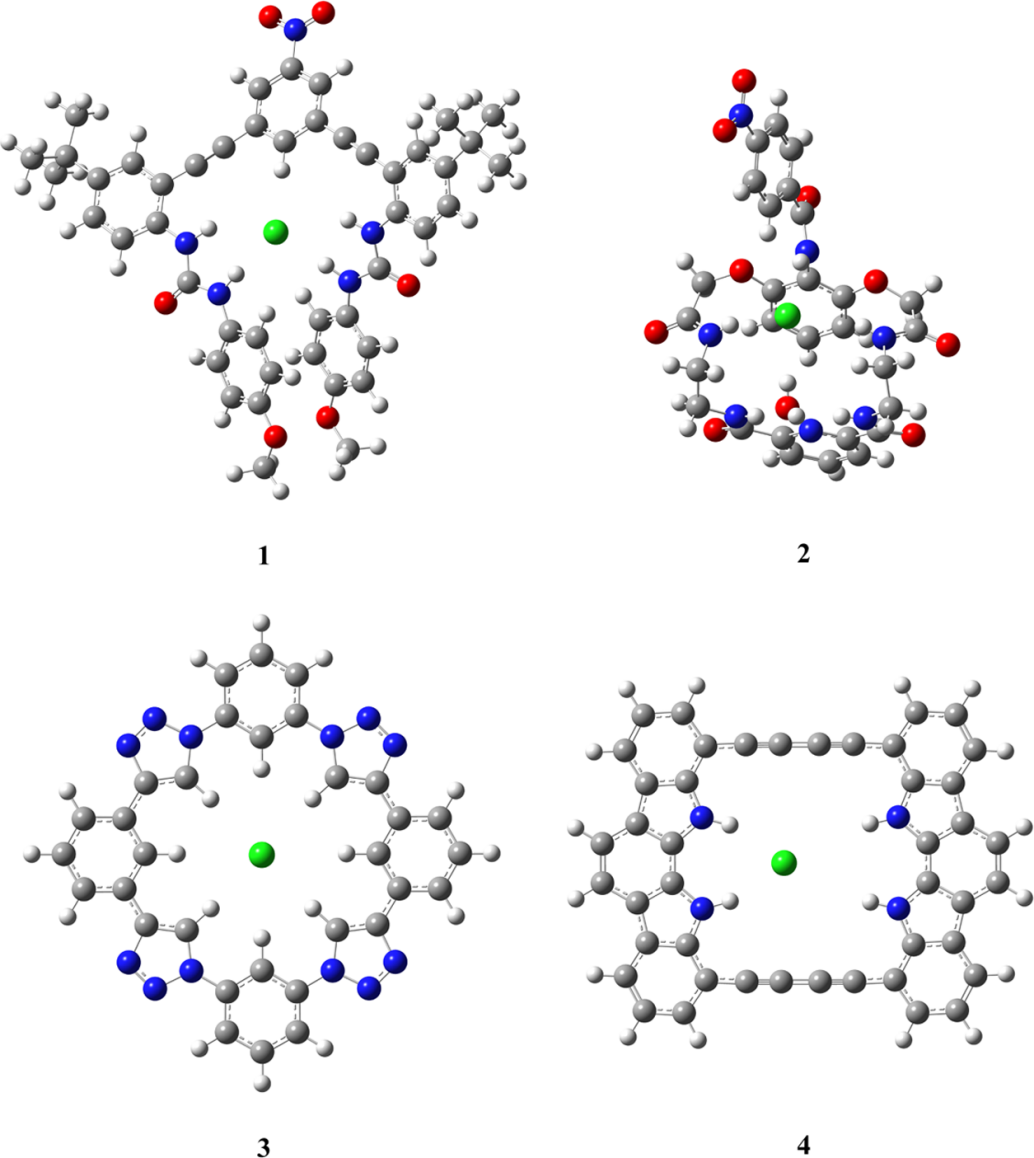
Main host–chloride frameworks used in the present work.

The question of how the solvent polarity affects host–guest interactions with chloride being a guest has been addressed in the past.^5−8^ Most recently, linear free energy dependence on E_T_(30) has been demonstrated^9^ for the bis(arylethynyl phenylurea) host, **1**. Within this framework, chloride interacts with the host via four N–H···Cl hydrogen bonds and with one C–H bond. Another approach to the analysis of noncovalent host–chloride interactions has been reported by Jurczak and co-workers^10^ who studied the influence of cryptand substituents, spanning from *tert*-butyl (**2**) to the nitro (**2a**) group. In this case, chloride is held by three N–H···Cl hydrogen bonds and additionally one hydrogen bond to a water molecule when experiments are carried out in a dimethyl sulfoxide (DMSO)–water mixed solvent. Two other host–guest frameworks pertinent to studies presented in this contribution involve chloride anion interactions exclusively with hydrogen from either C–H or N–H. The first one is represented by the triazolophane tetraphenylene macrocycle, **3**, for which the studies of electrostatic contribution to binding energy as a function of the distance have been reported.^5,8^ It comprises eight C–H···Cl bonds symmetrically directed toward the chloride anion, with C–H coming alternately from benzene and triazole rings. Indolocarbazole macrocycle, **4**, serves as an example of interactions with N–H protons only. Representative host–guest arrangements, used in the present studies, are illustrated in Figure 1.

For a long time,^11^ the isotopic fractionations of chlorine have been used for elucidation of mechanisms associated with chemical and enzymatic transformations in biochemical^1−3^ and environment-oriented systems.^12−14^ However, the theoretical evaluation of heavy-atom (i.e., carbon and heavier elements) isotope effects is hampered by the required high precision of calculations^15^ since these isotope effects are very small.^16,17^ The largest chlorine kinetic isotope effects are expected not to exceed 2% (when expressed as the percentage deviation from unity).^18^ The calculations of isotope effects on equilibrium processes, like enzymatic binding or host–guest interactions, are even harder because these isotope effects are smaller than those arising from the kinetic processes. Therefore, only scarce data on isotopic fractionation on noncovalent interactions^19^ (e.g., encapsulation) are available and predominantly connected with deuterium isotope effects,^20−23^ which are much larger than those of heavy atoms. We have evaluated the chlorine isotope effect on the binding step of a haloacid dehalogenase^24^ catalyzed reaction and showed that it can amount to about 25% of the apparent chlorine isotopic fractionation. This shows that these small isotope effect may be crucial to the interpretation of mechanisms of enzymatically and environmentally important reactions. Also, experimental determination of isotope effects may be severely affected by isotopic fractionation during analytical methodology,^25,26^ such as liquid chromatography.^27^

In this contribution, we have used the recently published data on the hydrogen-bonded systems in which the organic frame interacts with the chlorine anion via hydrogen bonds to C–H N–H groups, to evaluate the influence of the environment on the isotopic fractionation of chloride and at the same time to critically evaluate the computational protocol of the calculations of small isotopic fractionation associated with noncovalent interactions.

## Theoretical Methods

**Figure 2:**
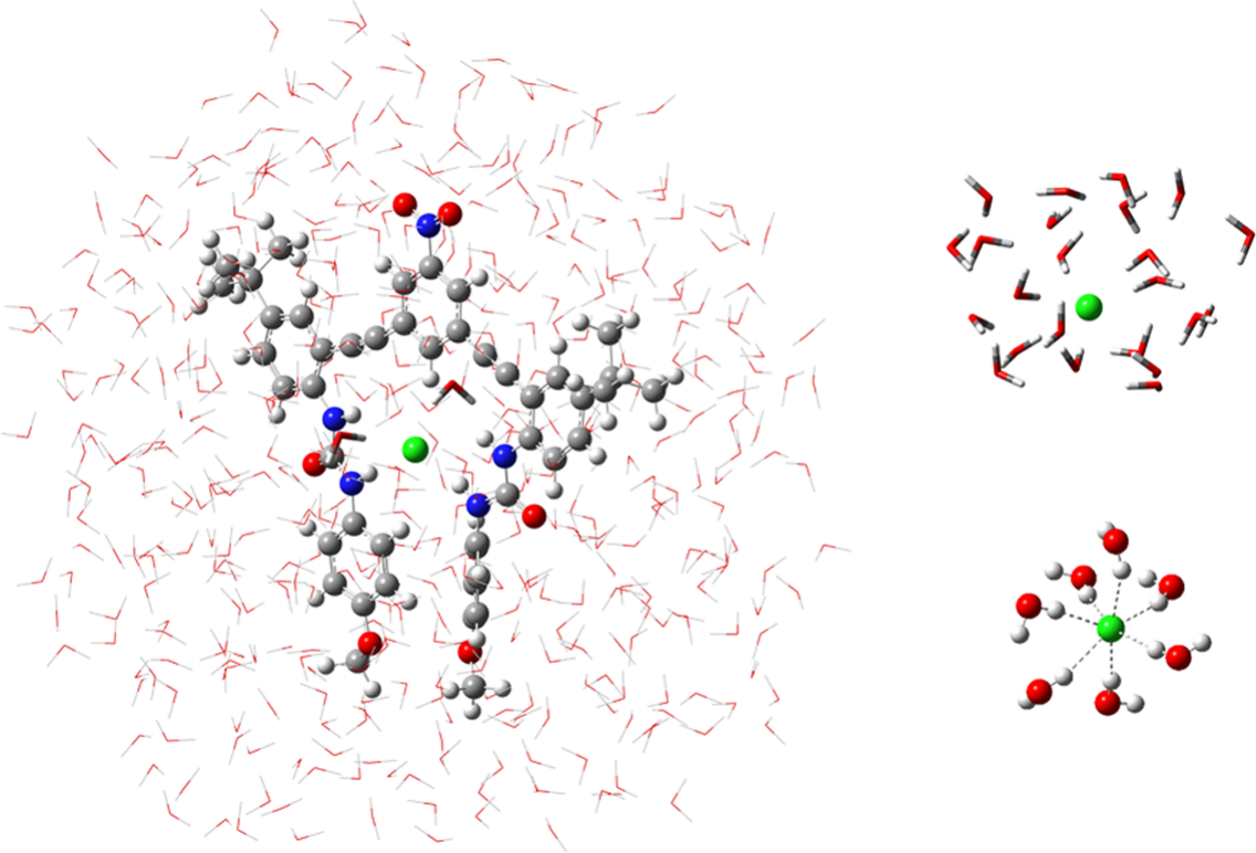
QM/MM models of host–guest complex **1** (a: left) and chloride (b: upper right). The QM region rendered as balls and sticks and the MM region rendered with a wireframe (for clarity) with two water molecules hydrogen-bonded to chloride rendered as tubes in the left panel and the right panel. The hydrogen-bonding pattern of chloride and the first solvation shell in the aqueous solution (c: lower right).

The geometries of all considered host–guest structures were first optimized in the gas phase to the nearest energy minimum at the density functional theory (DFT) level of theory, using the ωB97X-D functional^28^ expressed in the def2-TZVP basis set^29^ as implemented in the Gaussian16 program.^30^ Vibrational analysis was used to ensure that the optimized geometry corresponded to a stationary point representing a minimum on the potential energy surface (3n-6 real vibrations). The counterpoise correction^31^ was used for the calculations of binding energies and basis set superposition error (BSSE). Subsequently, gas-phase structures were used as starting points for geometry optimizations and frequency calculations for complexes in solutions. Two models of the solvent environment were used. The first one involved a solvation model based on density (SMD) continuum solvent model,^32^ which used the bulk properties of the solvent to create a dielectric cavity in which the solute was immersed. Specific parameters for water, chloroform, dichloromethane, argon, *n*-octane, and DMSO were used where appropriate. In the second approach, explicit solvent molecules were used. Within this approach, we used the subtractive ONIOM protocol^33^ of quantum mechanics/molecular mechanics (QM/MM) calculations,^34^ in which different parts of the studied system were treated at different theory levels. This protocol was applied to host–guest complex **1**, host without a chloride anion, and a chloride anion, to model their behavior in the aqueous solution. In the case of **1**, the model was built by placing the complex in a cube with a side of 40 Å of explicit TIP3P^35^ water molecules and running Langevin dynamics^36^ for 10 ps at 300 K using periodic box conditions and the Amber force field^37^ as implemented in HyperChem.^38^ The same procedure was carried out for the host molecule (see Figure S1 in the Supporting Information). Upon completion of the simulation, all water molecules farther than 15 Å from the chloride were removed. This procedure yielded a structure illustrated in Figure 2a, which contained 466 water molecules in the model. The same DFT level as for the gas-phase calculations was used for the QM part. The MM parameters of the Amber force field as implemented in Gaussian were used. Partial atomic charges were assigned using the Qeq method.^39^ No constraints on the optimization were imposed. The model of an explicitly solvated host was obtained from the optimized structure of the solvated host–guest complex by removing the chloride anion and repeating the calculations. The model of the chloride anion in an explicit water solution was obtained in an analogous way using a radius of 5 Å from the ion, resulting in the inclusion of 23 water molecules as presented in Figure 2b.

Chlorine equilibrium isotope effects, ^37^Cl-EIE, were calculated at 298 K using the Isoeff program^40^ from harmonic frequencies for isotopic species following the Bigeleisen equation.^41^

## Results and Discussion

Since chlorine equilibrium isotope effects, ^37^Cl-EIE, are very small, the results are presented as isotopic fractionation factors, ε, which express isotope effects as the deviation from unity in “per-mil” units [‰] (that correspond to mUr of the SI system)
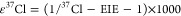
1In this notation, negative values correspond to isotope effects larger than unity (so-called normal isotope effects), while positive values correspond to isotope effects smaller than unity (so-called inverse isotope effects).

To study the contribution of the host–guest interactions on the chlorine isotopic fractionation, we have studied the hypothetical process of transferring the isolated chloride anion from the gas phase into the host environment. As the single ion does not contribute to vibrational analysis, the sole contribution to the calculated isotopic effects originates in the changes of vibrations upon isotopic substitution in the host–guest system. It should be kept in mind that these values would be markedly different if the solvated chloride was considered. For example, for the process of transferring the isolated chloride anion from the gas phase to the aqueous solution, where it forms a network of strong hydrogen bonds with the first solvation shell (illustrated in Figure 2c), the corresponding isotope effect calculated at the QM/MM level using the model presented in Figure 2b is 0.9956 (an isotopic fractionation factor of 4.4‰). These contributions to the overall isotopic fractionation vary with the solvent and would render the interpretation of the influence of host–guest interactions difficult.

Theoretical predictions of heavy-atom isotope effects pose numerous problems since they are very small. Therefore, the reported experimental structural results provided us with a unique opportunity to evaluate the quality of different theoretical models used in quantum-chemical calculations of isotope effects associated with changes in weak interactions. We have resorted to the theory level, which proved successful in our recent studies on carbon vapor pressure isotope effects of ethanol,^42^ carbon isotope effects on adsorption on graphene,^43^ and isotope effects of oxygen and sulfur in phosphates.^44^ This level has also been used recently for studies of over 12 000 chemical reactions^45^ and thus provides an excellent reference level for future studies. BSSE correction has been applied for gas-phase complexes. However, no counterpoise correction for ONIOM and continuum solvent models is available in the used software.

We have attempted to quantify the influence of a few environmental effects on chlorine isotope effects on host–guest interactions. For complex **1**, literature data indicate the linear dependence of the complexation energy on the polarity of the solvent. We have, therefore, optimized the structure of this complex for pure solvents used in the experiments (chloroform, dichloromethane, DMSO, and water) using the SMD continuum solvent model, as well as in the gas phase and low-polarity environments of argon and *n*-octane. It should be noted that the inclusion of the counterpoise correction influences slightly the obtained results. For consistency, we compare only the uncorrected results. To illustrate the influence of the counterpoise correction, the corresponding values are reported in parentheses in the first line of Table 1. Additionally, we have used this host–guest complex to compare the results obtained with explicit and implicit solvent models for the aqueous solution. The obtained results are summarized in the first eight lines of Table 1.

**Table 1:** Chlorine Isotopic Fractionation Factors, ε, Calculated for Different Host–Guest Systems under Different Conditions

			*d*_XH···Cl_ [Å]			
complex	solvent	structural feature	X = N	X = C	X = O	ε^37^Cl [‰]
**1**	none		2.15(2.19)	2.33(2.35)		2.27(2.13)
**1**	Ar(SMD)		2.20	2.35		2.14
**1**	*n*-octane(SMD)		2.21	2.37		1.88
**1**	CHCl_3_(SMD)		2.23	2.43		1.80
**1**	CH_2_Cl_2_(SMD)		2.25	2.47		1.63
**1**	DMSO(SMD)		2.26	2.48		1.63
**1**	water(SMD)		2.26	2.48		1.63
**1**	water(QM/MM)		2.31	2.58	2.41	1.77
**2**	DMSO(SMD)	–NO_2_	2.56		2.12	1.75
**2a**	DMSO(SMD)	–*t*Bu	2.54		2.11	1.74
**2**	DMSO(SMD)	–NO_2_	2.43			1.28
**2a**	DMSO(SMD)	–*t*Bu	2.43			1.21
**3**	water(SMD)			2.68		0.49
**3**	none			2.61		0.96
**3a**	none			2.45		1.31
**3b**	none			2.86		0.34
**4**	water(SMD)	NH/NH	2.32			1.13
**4**	none	NH/NH	2.35			0.77
**4se**	none	Se/Se	2.16			1.62
**4s**	none	S/S	2.14			1.73
**4o**	none	O/O	2.09			1.90

Considering the results for the gas phase and continuum solvent models, a slight dependence on the distance of chloride from hydrogens N–H and C–H can be noticed, especially in the environment of low polarity. The isotopic fractionation drops from 2.3‰ for the gas phase to around 1.6‰ for DMSO. Further increase in polarity does not introduce any changes. Table 1 lists averaged distances for hydrogen-bonding contacts, i.e., for arrangements with *d*_X···Cl_ less than about 3.6 Å. Therefore, in the case of host–guest complex **1**, distances to only two out of four N–H groups are included.

The comparison of the results obtained with different solvent models exposes, not surprisingly, the weakness of continuum solvent models that neglect explicit interactions between the solute and solvent molecules in the first solvation sphere. The structure of **1** optimized using the QM/MM protocol (Figure 2a) indicates that two water molecules located perpendicularly to the plane of the complex are in the hydrogen-bonding contact with chloride. This is manifested in the increase of ε^37^Cl, although the increase is surprisingly small, i.e., about 0.3‰. The structural differences of the complex obtained with these models are also worth noting. In the structures obtained in the gas phase and using the SMD model of the solvent, the “bottom” (as presented in Figure 1) benzene rings are stacked, while in the one obtained in QM/MM calculations, one of these rings is in the plane of the complex, while the other is twisted away from this plane, roughly in the perpendicular arrangement, leading to weaker interactions with C–H and N–H fragments.

**Figure 3:**
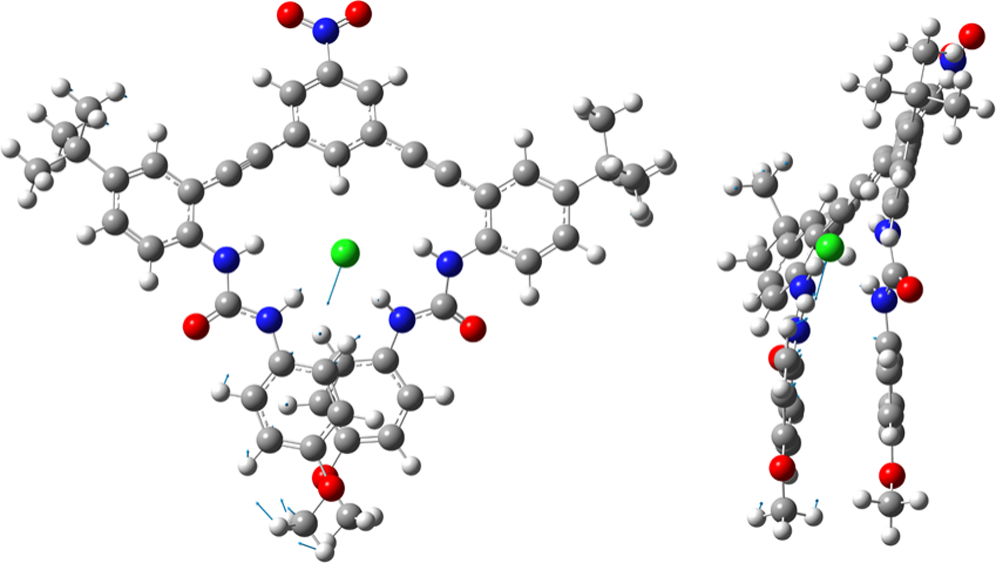
Translation vector of the most isotope sensitive normal mode of host–guest complex **1**.

The vibrational analysis of isotopic frequencies performed within the harmonic approximations indicates that only a limited number of normal modes contribute to the observed isotopic fractionations, most of which are contained in the range of 150–250 cm^–1^ and correspond to the vibrations that result in the movement of the chloride anion relative to the host frame, as illustrated by the translation vector for the most isotopically sensitive frequency at 156.3 cm^–1^ of the model of host–guest complex **1** in the gas phase in Figure 3.

The dependence on the distance of chloride from hydrogen atoms in N–H extends to host–guest complex **2**, which has been studied experimentally and therefore modeled herein in DMSO. The three N–H···Cl hydrogen bonds are slightly weaker than in the case of complex **1** with an averaged length of 2.43 versus 2.26 Å, which results in a smaller ε^37^Cl value of about 1.25‰. However, traces of water were present in experiments, resulting in the participation of one water molecule in the complex as evidenced by crystallography. When this water molecule is included in calculations, chlorine isotopic fractionation increases to about 1.75‰ despite the elongation of the N–H···Cl distance. On the other hand, the substituent in the para-position of the benzene ring has little influence on the isotopic fractionations, although significantly different substituents (nitro and *tert*-butyl groups) regarding the bulkiness and electronic effects were considered.

**Figure 4:**
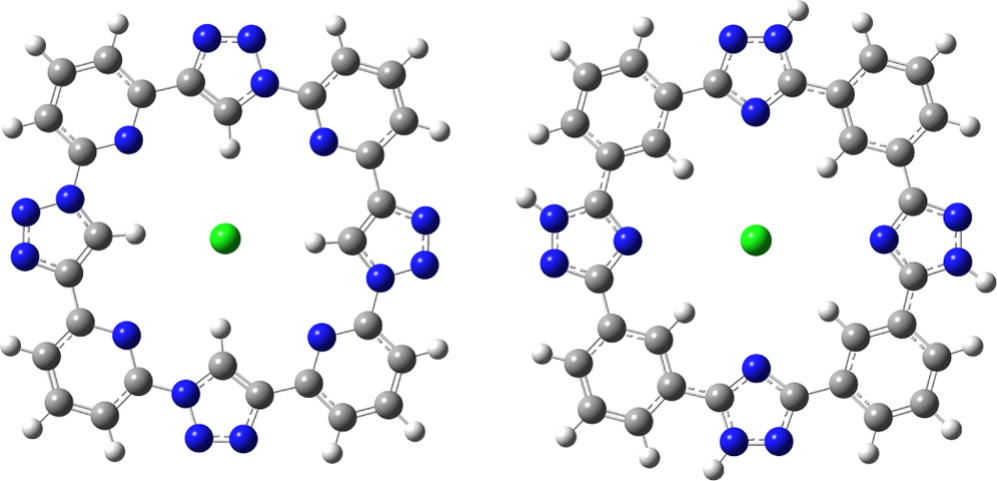
Structure of host–guest complexes **3a** (left) and **3b** (right).

In complex **1**, dominating interactions leading to the chlorine isotopic fractionation are undoubtedly those between chloride and N–H groups, but the picture is a bit obscured by the simultaneous presence of C–H···Cl interactions. We have attempted to separate the influence of these two interactions by considering two types of complexes in which chloride is involved in only one type of interaction, either with C–H or with N–H. As reported in the literature, complex **3** provided us with the opportunity to study isolated C–H···Cl interactions. Out of eight C–H bonds pointing toward chloride, only four were in weak hydrogen-bonding contact. Thus, not surprisingly, the value of the isotopic fractionation is quite low, under 1‰. Furthermore, we took advantage of the possibility of the computational separation of the influence of interactions of short C–H···Cl bonds from those operating on the longer distance by studying chlorine isotopic fractionation in complexes **3a** and **3b**, containing only short or long bonds, respectively (see Figure 4). Additionally, we have performed calculations for the structure isomeric to **3** that has a 4-fold symmetry axis perpendicular to the plane of the complex rather than two planes of symmetry. This isomer did not generate significant changes in C–H···Cl bonding, and consequently, the obtained ε^37^Cl values for these two compounds are practically identical (data not shown).

**Figure 5:**
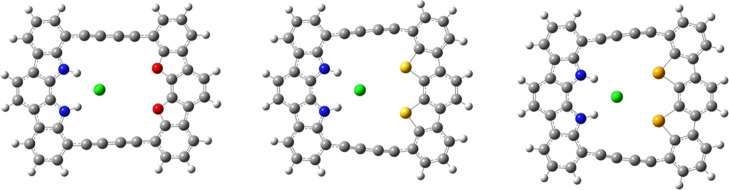
Modified structures of **4(O, S, Se)** with two NH groups replaced by two oxygen, sulfur, or selenium atoms (left to right).

Host–guest complex **4** has been used as an example of the sole presence of interactions with N–H bonds. In this complex, only two out of four hydrogen atoms in the N–H bond are hydrogen-bonded with the chlorine atom. Interestingly, opposite to complex **1**, in this case, a larger ε^37^Cl value of 1.13‰ has been obtained for the aqueous solution compared to the results in the gas phase. This is the result of slightly longer and thus weaker N–H···Cl bonds in the gas phase. We have carried out further studies of the influence of these hydrogen bonds on the chlorine isotopic fractionation by changing the two remote N–H groups to oxygen, sulfur, and selenium atoms. The structures of these complexes are presented in Figure 5. The obtained results conclude the data reported in Table 1.

We have found a linear correlation between the N–H···Cl hydrogen bond length and the chlorine isotopic fractionation. In fact, when only the gas-phase results are considered, the correlation is nearly perfect (*R* greater than 0.999), but even with the inclusion of the result obtained for the aqueous solution, it is still excellent. Similarly, a good correlation (*R* = 0.998) has been observed for the results obtained for host–guest complex **1**, as well as for complexes with varying C–H···Cl bond distances (*R* = 1.000 for the gas-phase results and 0.943 when one result that includes the continuum solvent model is added), although the slope is slightly different. This kind of correlation opens avenues for quantification and a priori predictions of small isotopic fractionations, although more data, both experimental and theoretical, are needed to obtain reliable results for broad types of host–guest complexes.

## Conclusions

**Figure 6:**
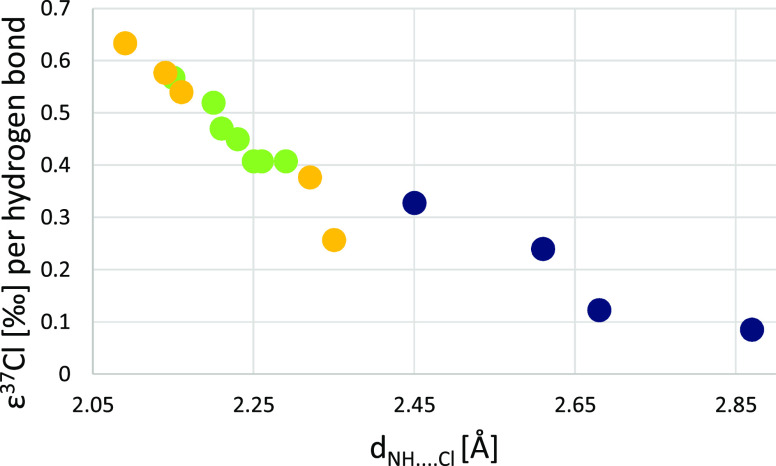
Dependence of chlorine isotopic fractionation on the X–H···Cl hydrogen bond length (green and orange circles: X = N, blue circle X = C).

Probably the most interesting result of our studies is identified as a nearly linear correlation between chlorine isotopic fractionation and the length of a hydrogen bond to chloride in host–guest complexes. It is presented in Figure 6, recalculated per single hydrogen bond. In a few cases with the presence of the O–H···Cl bond, the results were corrected for its much stronger isotopic fractionation (about 0.63‰ per position as estimated from the result obtained for the model of the chloride anion surrounded by water molecules, see Figure 2b). Their fit into the correlation indicates the additivity of the individual contributions to the apparent isotopic fractionation. Interestingly, even weak interactions with C–H hydrogen atoms show non-negligible contributions, although they are usually neglected and considered hidden in the precision of experimental determinations of chlorine isotopic composition.

Although the chlorine isotopic fractionations in weakly bonded systems are not large, our results indicate that they may have bearings on some seemingly remote problems like the mechanistic interpretation of chlorine isotope effects on the host–guest association, e.g., the formation of Michaelis complexes in enzymatic reactions.^46^ Furthermore, their presence may influence the analytical procedures of preparing (e.g., environmental) material for measurements. Extraction from nonpolar to polar solvents in measurement procedures can result in isotopic fractionation that may obscure interpretation, especially in environmental studies where small changes are traced.

## Supporting Information Available

Coordinates of optimized host–guest complexes **1**–**4** and conditions of MD simulations (PDF)
