# Supplementary material for: Isotopic Consequences of Host–Guest Interactions; Noncovalent Chlorine Isotope Effects
Source: J Phys Chem B. 2021 Feb 11;125(7):1874–80. doi: 10.1021/acs.jpcb.0c10691 (PMC8023698; doi:10.1021/acs.jpcb.0c10691)
Supplement: Supplementary file 1 — jp0c10691_si_001.pdf [file jp0c10691_si_001.pdf]

Isotopic Consequences of Host-guest Interactions; Noncovalent Chlorine Isotope Effects

Agata Paneth[1] and Piotr Paneth[2]*

1. *Department of Organic Chemistry, Faculty of Pharmacy, Medical University of Lublin, Chodźki 4a, 20-093 Lublin, Poland*
2. *Institute of Applied Radiation Chemistry, Faculty of Chemistry, Lodz University of Technology, Żeromskiego 116, 90-924 Lodz, Poland,*

Contents

1. Cartesian coordinates of the gas phase structures of host-guest complexes **1, 3,** and **4**,
2. Cartesian coordinates of the structures of host-guest complexes **2** in the continuum DSMO model,
3. Figure S1. Illustration of the Langevin dynamics calculations,
4. Cartesian coordinates of the QM/MM structures of host-guest complexes **1**.

Cartesian coordinates of the gas phase structures of host-guest complexes **1, 3,** and **4**

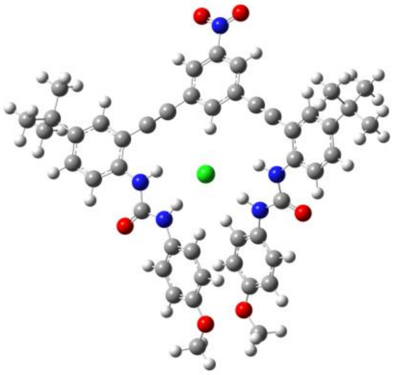

1

C	7.07909400	-1.96090900	-0.89487500
C	7.08682600	-0.56942400	-0.87063300
C	5.95383900	0.17361200	-0.58902300
C	4.74082100	-0.44950200	-0.30544900
C	4.71404500	-1.85880300	-0.31227400
C	5.86962300	-2.57755200	-0.61179100
C	8.31454500	-2.80647000	-1.20913000
C	9.54531900	-1.94373200	-1.49659500
C	8.63480900	-3.71307400	-0.01102400
C	8.04085500	-3.67659800	-2.44515800
N	3.57125900	0.24960900	-0.04069800
C	3.49303600	1.54115800	0.46549000
O	4.47461200	2.20421700	0.74860500
N	2.20493100	1.95236500	0.62200200
C	1.77473800	3.19402600	1.11226200
C	0.45890800	3.28013400	1.57348700
C	-0.06705700	4.48001900	1.99775600
C	0.71310700	5.63059800	1.97970700
C	2.02654200	5.55167100	1.54602100
C	2.55477800	4.34066900	1.11281100
O	0.10457800	6.78269600	2.39095200
C	0.86061900	7.96233100	2.34801100
C	3.51919000	-2.56825900	-0.00008000
C	2.51267600	-3.16756600	0.27328600
C	1.28291900	-3.83147600	0.55918400
C	1.26012200	-5.13349500	1.05497800
C	0.08482300	-3.15928400	0.32560400
C	-1.13928300	-3.77421400	0.57727700
C	-1.16727500	-5.07626700	1.07522500
C	0.03367500	-5.72179000	1.30117600
C	-2.34869800	-3.07046400	0.30284400
C	-3.36394000	-2.48073500	0.03911900
C	-4.60996600	-1.86606700	-0.27174100
C	-4.70992500	-0.50784800	-0.62347200
C	-5.98657200	-0.01656200	-0.92206600
C	-7.09296000	-0.83567100	-0.86221300
C	-7.01633100	-2.18482700	-0.51125300
C	-5.75592900	-2.67011700	-0.22502200
C	-8.27861600	-3.04527800	-0.46062000
C	-7.97781500	-4.48996700	-0.05644500
C	-9.25915100	-2.45461200	0.56387200
C	-8.94179500	-3.06297400	-1.84620700
N	-3.56323500	0.27190200	-0.68560100
C	-3.51923800	1.66209600	-0.77954000
O	-4.51447300	2.36215700	-0.72358300
N	-2.25436600	2.13292500	-0.93770800
C	-1.86136000	3.47928200	-0.99384400
C	-0.57354800	3.74925700	-1.46614900
C	-0.07764200	5.03390000	-1.47567600
C	-0.85883900	6.09082900	-1.02153300
C	-2.14553500	5.83683100	-0.57612500
C	-2.64453300	4.53893500	-0.56012100
O	-0.27812300	7.32690200	-1.03904700
C	-1.03729600	8.40282100	-0.55784800
N	0.00520300	-7.09682200	1.82351700
O	1.06616800	-7.65592300	2.00879400
O	-1.07775800	-7.59943000	2.04083800
H	7.99766500	-0.02625500	-1.08341800
H	6.00197800	1.25005200	-0.57429800
H	5.79148600	-3.65762100	-0.60731700
H	10.40002600	-2.58759300	-1.71557600

H	9.38914400	-1.29351700	-2.35970600	
H	9.80960000	-1.31998300	-0.64034700	
H	9.51707100	-4.32406900	-0.22036700	
H	8.83337500	-3.11642600	0.88143700	
H	7.80697000	-4.38642500	0.21526500	
H	8.91656500	-4.28633500	-2.68360700	
H	7.19783600	-4.34969700	-2.28353700	
H	7.80909100	-3.05382300	-3.31138100	
H	2.69819400	-0.24497000	-0.18085300	
H	1.46550600	1.33247000	0.28512200	
H	-0.16434700	2.39372200	1.56036400	
H	-1.09770400	4.54761400	2.31891300	
H	2.65937100	6.42725900	1.51709100	
H	3.57654600	4.28956600	0.77168200	
H	0.20244400	8.76239300	2.68426100	
H	1.72922400	7.91183700	3.01444300	
H	1.20428800	8.18169600	1.33127000	
H	2.17286400	-5.67690900	1.24564300	
H	0.10393600	-2.14623300	-0.06437700	
H	-2.10073600	-5.57796500	1.27968800	
H	-6.09376700	1.02062800	-1.18996100	
H	-8.05350100	-0.39566400	-1.10452700	
H	-5.60887800	-3.70461300	0.05111500	
H	-8.90626200	-5.06474100	-0.03283800	
H	-7.30443300	-4.97522200	-0.76572600	
H	-7.52719300	-4.54533000	0.93647000	
H	-10.17051500	-3.05689800	0.61038200	
H	-8.81010000	-2.43307500	1.55871500	
H	-9.54415600	-1.43406600	0.30423700	
H	-9.84948900	-3.67222800	-1.82662900	
H	-9.21870900	-2.05910200	-2.17141400	
H	-8.26331100	-3.48085300	-2.59246600	
H	-2.67540700	-0.21192300	-0.61909800	
H	-1.49033600	1.45534000	-1.00081300	
H	0.05259800	2.92657400	-1.79124600	
H	0.93317300	5.23142500	-1.80690700	
H	-2.77945200	6.63335000	-0.21338500	
H	-3.64440200	4.35287600	-0.20271500	
H	-0.40111100	9.28413800	-0.62798500	
H	-1.93822800	8.56476700	-1.16076000	
H	-1.32969500	8.25081400	0.48663400	
Cl	0.11921200	0.04014400	-0.85986800	
C	-6.04450500	1.19192900	0.13950700	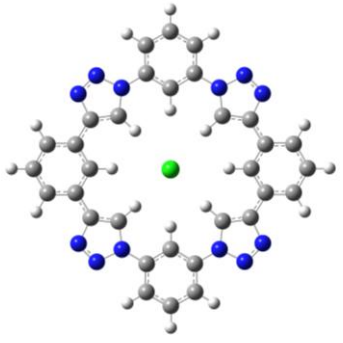 3
C	-4.66329000	1.18204200	0.00801700	
C	-3.96058200	-0.00982300	-0.07230600	
C	-4.65713600	-1.20523100	0.00816500	
C	-6.03830700	-1.22210600	0.13970200	
C	-6.71658800	-0.01682800	0.19607600	
N	-3.94931500	2.40216100	-0.03948100	
N	-3.70293900	4.50816600	-0.15329700	
C	-2.46408000	3.95276500	-0.05075000	
C	-2.61505000	2.59341900	0.02392100	
N	-4.59161900	3.57773000	-0.14648700	
C	-1.22166900	4.72594300	-0.02885300	
C	-1.22097000	6.11831900	-0.03160800	
C	-0.01692600	6.80462900	-0.00253500	
C	1.19054000	6.12437300	0.02799600	
C	1.19815200	4.73205700	0.02863600	
C	2.44442200	3.96513900	0.05129000	
C	2.60195300	2.60671700	-0.02433700	
N	3.93715600	2.42178600	0.03998500	
N	3.68042000	4.52653900	0.15495000	
C	4.65722300	1.20523100	-0.00733000	
C	3.96061700	0.00982500	0.07275700	

C	4.66336600	-1.18203500	-0.00733800	
C	6.04462700	-1.19191000	-0.13829500	
C	6.03845400	1.22211100	-0.13828300	
C	6.71675100	0.01684300	-0.19450200	
N	3.94939700	-2.40216100	0.03986600	
C	2.61517300	-2.59344900	-0.02425300	
C	2.46415400	-3.95278200	0.05057800	
N	3.70296500	-4.50816400	0.15386700	
N	4.59163300	-3.57771800	0.14749000	
C	1.22175600	-4.72596100	0.02803200	
C	-1.19806200	-4.73205500	-0.02933200	
C	-1.19040900	-6.12437100	-0.03059200	
C	0.01707500	-6.80464100	-0.00098800	
C	1.22110400	-6.11833900	0.02888000	
C	-2.60182000	-2.60670000	0.02441900	
N	-3.93706200	-2.42178000	-0.03932900	
N	-4.57363300	-3.60036500	-0.14685300	
C	-2.44434400	-3.96513100	-0.05123400	
N	-3.68038200	-4.52653900	-0.15431000	
N	4.57367200	3.60036600	0.14801900	
H	-6.56829400	2.13426600	0.19589300	
H	-2.88497800	-0.00688000	-0.19888800	
H	-6.55705200	-2.16724100	0.19617300	
H	-1.89445500	1.79165300	0.11998600	
H	-2.16352900	6.64936800	-0.05530400	
H	2.13046700	6.66014600	0.05016300	
H	1.88508300	1.80181000	-0.12161600	
H	2.88495700	0.00689500	0.19880500	
H	6.56844000	-2.13424100	-0.19453900	
H	6.55721700	2.16725300	-0.19442500	
H	1.89465900	-1.79170600	-0.12086900	
H	-2.13032200	-6.66013400	-0.05351600	
H	2.16368400	-6.64938800	0.05171900	
H	-1.88485300	-1.80179500	0.12133500	
C	-0.01005800	4.04803400	0.00092100	
C	0.01012900	-4.04804100	-0.00060600	
H	-0.00735600	2.96364400	0.00249700	
H	0.00738300	-2.96365400	-0.00048400	
H	0.01978500	-7.88797300	-0.00115200	
H	7.79438600	0.01958000	-0.29637500	
H	-0.01961700	7.88796000	-0.00383600	
H	-7.79418100	-0.01957200	0.29838600	
Cl	-0.00080400	0.00000200	0.00003400	
C	-0.04133600	6.12900700	0.00024600	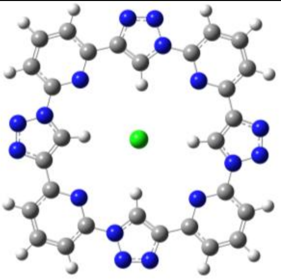 **3a**
C	-0.25113700	4.75193500	0.00002500	
C	1.98280400	4.34997100	0.00005500	
C	2.31353100	5.69887700	0.00029600	
C	1.26023900	6.59439600	0.00038700	
N	-3.75764300	4.29969700	-0.00002600	
C	-2.02591100	2.89599200	-0.00019500	
N	-2.69656900	5.01729300	0.00012400	
C	-4.35007400	1.98278000	-0.00023200	
C	-5.69897700	2.31354200	-0.00035700	
C	-6.59452400	1.26027400	-0.00012900	
C	-6.12916900	-0.04131600	0.00012800	
C	-4.75210600	-0.25114800	0.00008200	
C	-4.20271200	-1.60438800	0.00014400	
C	-2.89612800	-2.02584100	0.00018200	
N	-3.00896300	-3.37183000	0.00014700	
N	-5.01739600	-2.69659700	0.00002500	
C	-1.98280400	-4.34997000	0.00008700	
C	0.25113700	-4.75193400	0.00011200	
C	0.04133600	-6.12900600	-0.00003300	
C	-2.31353100	-5.69887700	-0.00007600	

C	-1.26023800	-6.59439500	-0.00013200	
C	2.02591200	-2.89599100	0.00019500	
N	3.75764200	-4.29969900	0.00007500	
N	2.69656700	-5.01729300	-0.00000300	
C	4.35007500	-1.98278200	0.00009600	
C	4.75210400	0.25114700	-0.00020400	
C	6.12916700	0.04131700	-0.00042900	
C	6.59452400	-1.26027400	-0.00027300	
C	5.69897800	-2.31354300	0.00003100	
C	2.89612500	2.02584100	-0.00016800	
N	3.00896200	3.37182900	-0.00004900	
N	4.29974100	3.75763800	0.00009800	
C	4.20270900	1.60438700	-0.00016400	
N	5.01739400	2.69659500	0.00001600	
N	-4.29974100	-3.75763900	0.00001400	
H	-0.89034600	6.79705800	0.00031700	
H	3.34595300	6.01096100	0.00042500	
H	-1.49100500	1.94613300	-0.00050200	
H	-6.01103000	3.34597400	-0.00051800	
H	-6.79722600	-0.89032200	0.00026900	
H	-1.94632100	-1.49085200	0.00033100	
H	0.89034600	-6.79705600	-0.00008000	
H	-3.34595300	-6.01096100	-0.00017600	
H	1.49101200	-1.94612600	0.00046300	
H	6.79722300	0.89032300	-0.00062500	
H	6.01103100	-3.34597400	0.00011500	
H	1.94631400	1.49085900	-0.00035100	
H	7.65922000	-1.45879600	-0.00039000	
H	-1.45873500	-7.65909600	-0.00027200	
H	-7.65922000	1.45879700	-0.00015600	
H	1.45873600	7.65909700	0.00058700	
Cl	0.00000700	0.00000100	-0.00007200	
N	-3.37189800	3.00889800	-0.00027400	
N	3.37189900	-3.00889900	0.00024300	
C	-1.60438900	4.20255300	-0.00007700	
C	1.60438800	-4.20255200	0.00016700	
N	3.88164700	-0.75680800	0.00002300	
N	0.75683900	3.88150400	-0.00006200	
N	-3.88164800	0.75680600	-0.00005700	
N	-0.75683900	-3.88150400	0.00016600	
C	1.90849600	5.86022100	-0.30339200	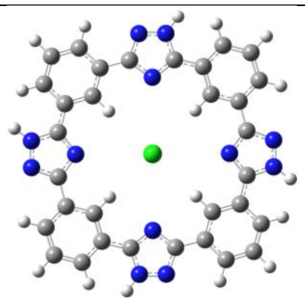 **3b**
C	1.22256200	4.65744500	-0.15388200	
C	1.91252200	3.44739400	-0.14058000	
C	3.30311300	3.46741800	-0.23279500	
C	3.98230900	4.67256300	-0.40253700	
C	3.28410300	5.86639700	-0.44441000	
N	-2.11975500	5.27974100	0.65549700	
N	-0.84400100	5.69864900	0.62687100	
C	-3.48882900	3.30730600	0.03331400	
C	-4.69388600	4.00267800	-0.04097000	
C	-5.89211300	3.31415400	-0.11512200	
C	-5.88889400	1.93180500	-0.12130700	
C	-4.68677300	1.23093400	-0.05445400	
C	-4.70157100	-0.23605600	-0.09704600	
N	-5.72579200	-0.91795500	-0.58178500	
C	-3.30298100	-3.46730100	0.23300400	
C	-1.91240000	-3.44738600	0.14072400	
C	-1.22254300	-4.65750600	0.15403600	
C	-1.90850500	-5.86025100	0.30359300	
C	-3.98221500	-4.67242100	0.40281800	
C	-3.28410500	-5.86631600	0.44468400	
N	2.11969700	-5.27943200	-0.65599700	
N	0.84395100	-5.69839800	-0.62738900	
C	3.48877500	-3.30722100	-0.03316200	

C	4.68670900	-1.23086800	0.05467700	
C	5.88882400	-1.93175100	0.12156900	
C	5.89204700	-3.31409600	0.11536500	
C	4.69381700	-4.00260700	0.04115600	
C	4.70157800	0.23612400	0.09724100	
N	5.72594000	0.91798100	0.58174300	
N	-5.30104200	-2.18551700	-0.46217800	
H	1.34879500	6.78652100	-0.30949000	
H	1.37917400	2.50035900	-0.02775200	
H	5.05981100	4.67770400	-0.52397300	
H	-4.69724800	5.08636200	-0.07446100	
H	-6.81648500	1.37792300	-0.18501900	
H	-1.37880900	-2.50048900	0.02775700	
H	-1.34885700	-6.78658500	0.30967300	
H	-5.05971000	-4.67750000	0.52432400	
H	6.81641800	-1.37787700	0.18531900	
H	4.69714900	-5.08629300	0.07461500	
C	-3.47562000	1.91364700	0.02468300	
C	3.47554300	-1.91355800	-0.02452000	
H	-2.52951900	1.36959000	0.07000700	
H	2.52941900	-1.36951600	-0.06995400	
H	6.82721100	-3.85676400	0.17828800	
H	-3.81335500	-6.80053800	0.58635400	
H	-6.82727800	3.85682300	-0.17801800	
H	3.81326700	6.80067500	-0.58603600	
Cl	-0.00014000	-0.00061600	-0.00095200	
C	-0.23060800	4.67586000	0.05495200	
C	0.23061300	-4.67589400	-0.05491800	
C	2.22701900	-4.04390700	-0.12342900	
C	4.05886500	2.22434400	-0.06548400	
C	-2.22705900	4.04397200	0.12349000	
C	-4.05873600	-2.22423900	0.06567200	
N	3.66509800	1.00042700	-0.31800400	
N	-1.04328800	3.64638500	-0.27452000	
N	-3.66511600	-1.00030800	0.31836000	
N	1.04330600	-3.64655400	0.27496000	
N	5.30126200	2.18557200	0.46213200	
H	5.84358200	2.93785500	0.84449200	
H	-2.81962100	5.82505400	1.12338700	
H	-5.84322000	-2.93781500	-0.84471100	
H	2.81952000	-5.82447600	-1.12426500	
C	3.93907300	2.85690200	0.00016200	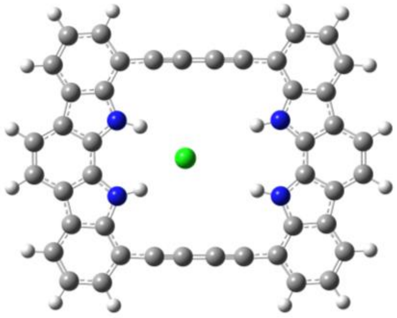 **4**
C	5.45319200	5.17316400	0.00011500	
C	5.34761000	2.78254500	0.00018800	
C	3.27625700	4.09191400	0.00009500	
C	4.06203500	5.24386200	0.00007600	
C	6.10340200	3.94970400	0.00016500	
H	3.57039000	6.20802200	0.00002700	
H	7.18540300	3.90108400	0.00018700	
C	5.68300300	1.37880900	0.00023200	
C	5.68296400	-1.37891100	0.00030900	
C	4.45951000	0.69659000	0.00024400	
C	6.90797300	0.68725300	0.00025500	
C	6.90795400	-0.68738900	0.00028900	
C	4.45949000	-0.69665700	0.00029300	
H	7.83926600	1.23956300	0.00024200	
N	3.41635000	1.59371000	0.00020700	
H	2.43036600	1.33269800	0.00019000	
N	3.41630600	-1.59374900	0.00031300	
H	2.43032800	-1.33271400	0.00037300	
H	7.83923100	-1.23972600	0.00030200	
H	6.02809300	6.08987000	0.00009800	
C	5.34753200	-2.78263800	0.00034600	
C	3.93899400	-2.85695600	0.00035500	

C	6.10329100	-3.94981800	0.00037100	
C	3.27614300	-4.09194900	0.00039900	
C	5.45304700	-5.17325900	0.00041000	
H	7.18529400	-3.90122800	0.00036200	
C	4.06188800	-5.24391800	0.00042400	
H	6.02792200	-6.08998200	0.00043000	
H	3.57021600	-6.20806500	0.00045800	
C	1.85947100	4.14769100	0.00003100	
C	0.65222500	4.15349100	-0.00002900	
C	-0.71192300	4.15394500	-0.00005300	
C	-1.91924200	4.15320500	-0.00009100	
C	1.85935500	-4.14768500	0.00042700	
C	0.65210900	-4.15346300	0.00041000	
C	-0.71204000	-4.15394400	0.00023200	
C	-1.91935800	-4.15322400	0.00007500	
C	-3.33639600	4.09837400	-0.00030800	
C	-4.12510900	5.24814500	-0.00043300	
C	-3.99846700	2.86384500	-0.00036100	
C	-5.51616500	5.17333000	-0.00059900	
H	-3.63623000	6.21365100	-0.00039000	
C	-5.40475700	2.78356800	-0.00051600	
C	-6.16387700	3.94869600	-0.00063900	
H	-6.09330600	6.08859400	-0.00068900	
H	-7.24565600	3.89738000	-0.00075900	
C	-3.33651000	-4.09835000	-0.00001600	
C	-3.99854700	-2.86380300	-0.00011300	
C	-4.12525600	-5.24809900	-0.00003900	
C	-5.40483400	-2.78348600	-0.00024600	
C	-5.51631000	-5.17324500	-0.00016300	
H	-3.63640400	-6.21361900	0.00003400	
C	-6.16398800	-3.94859300	-0.00026900	
H	-6.09347700	-6.08849300	-0.00018300	
H	-7.24576500	-3.89724700	-0.00037200	
N	-3.47339300	1.60047700	-0.00026900	
N	-3.47343700	-1.60045000	-0.00011300	
C	-4.51166900	0.69624700	-0.00033100	
C	-4.51168800	-0.69619000	-0.00025900	
H	-2.49160600	1.37200700	-0.00011600	
H	-2.49164400	-1.37200700	-0.00002600	
C	-5.73574000	-1.37818500	-0.00034700	
C	-6.95997300	-0.68723300	-0.00050500	
H	-7.89084300	-1.24005900	-0.00057100	
C	-5.73570200	1.37827600	-0.00048900	
C	-6.95995400	0.68735800	-0.00058000	
H	-7.89080900	1.24021000	-0.00070200	
Cl	0.53496400	0.00003600	0.00017900	
C	-4.07970300	2.78534800	0.00002900	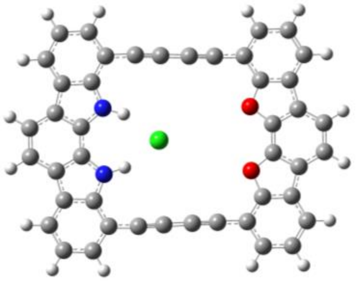 **4o**
C	-5.45920200	5.15140900	0.00009100	
C	-5.47522700	2.77161700	-0.00004400	
C	-3.32954100	3.95981000	0.00013300	
C	-4.06737700	5.14660900	0.00016300	
C	-6.17936000	3.96958300	-0.00001300	
H	-3.52420900	6.08221500	0.00024600	
H	-7.26191200	3.97717100	-0.00006900	
C	-5.84397700	1.37288700	-0.00014100	
C	-5.84406900	-1.37266900	-0.00029400	
C	-4.62691100	0.69188000	-0.00011700	
C	-7.06480700	0.68897300	-0.00024100	
C	-7.06485300	-0.68867400	-0.00031700	
C	-4.62695800	-0.69174200	-0.00019500	
H	-7.99616100	1.24039000	-0.00025700	
H	-7.99624400	-1.24002900	-0.00039500	
H	-5.98113900	6.09973000	0.00011900	

C	-5.47541000	-2.77142300	-0.00035200	
C	-4.07988700	-2.78524700	-0.00028400	
C	-6.17962300	-3.96934300	-0.00045300	
C	-3.32980300	-3.95975800	-0.00031200	
C	-5.45954300	-5.15121600	-0.00048100	
H	-7.26217500	-3.97685900	-0.00050700	
C	-4.06771800	-5.14650900	-0.00041100	
H	-5.98154300	-6.09950300	-0.00055800	
H	-3.52461100	-6.08215100	-0.00043500	
C	-1.91123500	3.96481700	0.00021400	
C	-0.70605200	3.99373800	0.00026300	
C	0.65720100	4.04039200	0.00019500	
C	1.86268600	4.09202300	0.00014700	
C	-1.91149800	-3.96486300	-0.00024100	
C	-0.70631500	-3.99383300	-0.00018500	
C	0.65694000	-4.04040700	-0.00014000	
C	1.86242700	-4.09199300	-0.00009900	
C	3.28075300	4.06517300	0.00022400	
C	4.05004700	5.22695300	0.00030200	
C	3.96321000	2.83845500	0.00021400	
C	5.44217600	5.17833100	0.00036600	
H	3.54166300	6.18263900	0.00031100	
C	5.37926800	2.78341600	0.00027900	
C	6.11091500	3.96404700	0.00035500	
H	6.00384000	6.10389600	0.00042500	
H	7.19430200	3.93614300	0.00040500	
C	3.28049600	-4.06524100	-0.00004300	
C	3.96303200	-2.83856800	0.00002700	
C	4.04971600	-5.22707100	-0.00005000	
C	5.37909200	-2.78361800	0.00008900	
C	5.44184800	-5.17853700	0.00001000	
H	3.54127200	-6.18272500	-0.00010300	
C	6.11066400	-3.96429500	0.00007900	
H	6.00345300	-6.10413700	0.00000200	
H	7.19405300	-3.93646000	0.00012600	
N	3.45709900	1.57901000	0.00014700	
N	3.45699900	-1.57909100	0.00004400	
C	4.50623000	0.69805200	0.00016600	
C	4.50618500	-0.69819700	0.00012100	
H	2.46460700	1.27343100	0.00009800	
H	2.46452700	-1.27347100	0.00002500	
C	5.72762000	-1.38413400	0.00015200	
C	6.95166400	-0.68703400	0.00022900	
H	7.88642000	-1.23456500	0.00025200	
C	5.72771000	1.38391100	0.00024500	
C	6.95170900	0.68673300	0.00027600	
H	7.88650000	1.23420300	0.00033600	
Cl	0.80688900	0.00008300	0.00003200	
O	-3.56277200	-1.52912900	-0.00018900	
O	-3.56267100	1.52919600	-0.00001500	
C	3.73358400	-3.11816500	0.00004300	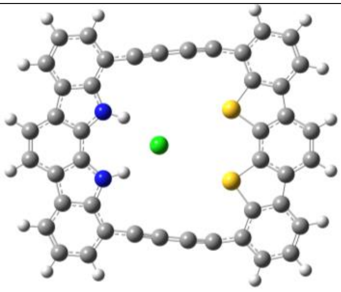 **4s**
C	5.55855400	-5.18133500	0.00016600	
C	5.10249500	-2.82471100	0.00002700	
C	3.25763500	-4.43745000	0.00012300	
C	4.19410400	-5.46426100	0.00018100	
C	6.01738200	-3.87704300	0.00009000	
H	3.84932800	-6.48991500	0.00024000	
H	7.08136800	-3.67368700	0.00007800	
C	5.32998000	-1.39403900	-0.00005900	
C	5.33021800	1.39344000	-0.00022800	
C	4.11902700	-0.69685400	-0.00010300	
C	6.54304700	-0.68767200	-0.00010100	
C	6.54316400	0.68686600	-0.00018400	
C	4.11914600	0.69646300	-0.00018800	

H	7.48064000	-1.22987700	-0.00006700	
H	7.48085100	1.22891100	-0.00021600	
H	6.26778900	-5.99928700	0.00021400	
C	5.10297800	2.82415200	-0.00031500	
C	3.73411700	3.11783800	-0.00033300	
C	6.01804400	3.87632800	-0.00037800	
C	3.25839200	4.43720400	-0.00041200	
C	5.55943700	5.18069800	-0.00045800	
H	7.08199500	3.67279100	-0.00036500	
C	4.19503500	5.46385600	-0.00047600	
H	6.26881100	5.99853000	-0.00050900	
H	3.85043400	6.48956800	-0.00053900	
C	1.84985500	-4.62863100	0.00012900	
C	0.64481300	-4.55430500	0.00011800	
C	-0.71240700	-4.42372200	0.00011900	
C	-1.90775900	-4.26028800	0.00012200	
C	1.85064500	4.62862500	-0.00043000	
C	0.64559500	4.55443500	-0.00040600	
C	-0.71161300	4.42372900	-0.00024000	
C	-1.90695800	4.26024200	-0.00009800	
C	-3.31837500	-4.11651700	0.00022100	
C	-4.13064600	-5.25121700	0.00030800	
C	-3.95301300	-2.86202900	0.00022400	
C	-5.51875500	-5.16289600	0.00039600	
H	-3.65058500	-6.22131700	0.00030400	
C	-5.36692700	-2.77545300	0.00031300	
C	-6.14206200	-3.92680700	0.00039900	
H	-6.10914200	-6.07022500	0.00046100	
H	-7.22338000	-3.85581800	0.00046700	
C	-3.31760200	4.11674900	-0.00001300	
C	-3.95247600	2.86238200	0.00006000	
C	-4.12965900	5.25160300	0.00000200	
C	-5.36640600	2.77607200	0.00014900	
C	-5.51778500	5.16354300	0.00008800	
H	-3.64941500	6.22161200	-0.00005500	
C	-6.14132500	3.92757200	0.00016200	
H	-6.10800100	6.07098300	0.00009800	
H	-7.22265600	3.85678700	0.00023000	
N	-3.42555800	-1.60583800	0.00015200	
N	-3.42525700	1.60609100	0.00006200	
C	-4.46028700	-0.70043500	0.00019300	
C	-4.46015500	0.70088200	0.00015200	
H	-2.43224300	-1.31897500	0.00008100	
H	-2.43199600	1.31904500	0.00000800	
C	-5.68953800	1.37480600	0.00021000	
C	-6.91534200	0.68596200	0.00030700	
H	-7.84683900	1.23877000	0.00034900	
C	-5.68979600	-1.37412700	0.00029100	
C	-6.91547100	-0.68505300	0.00034700	
H	-7.84707100	-1.23768500	0.00042300	
Cl	-0.74609300	-0.00012700	-0.00006400	
S	2.71554200	-1.71614800	-0.00004300	
S	2.71583500	1.71599600	-0.00025100	

C	3.44326400	-3.22473400	0.00002600	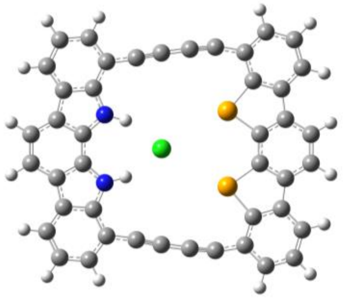 **4se**
C	5.38683500	-5.17545100	0.00011600	
C	4.78863500	-2.84412000	0.00001800	
C	3.05216500	-4.57049000	0.00008000	
C	4.04420900	-5.54390300	0.00012300	
C	5.76213700	-3.84332100	0.00006400	
H	3.76264000	-6.58880100	0.00016300	
H	6.81268200	-3.57875700	0.00005900	
C	4.97125200	-1.40056200	-0.00004000	
C	4.97174700	1.39932400	-0.00015600	
C	3.76485500	-0.69533300	-0.00007600	
C	6.18016600	-0.68874300	-0.00006300	
C	6.18040900	0.68707700	-0.00012000	
C	3.76510100	0.69452100	-0.00013400	
H	7.12060600	-1.22625200	-0.00003500	
H	7.12104000	1.22425400	-0.00013700	
H	6.14758100	-5.94581200	0.00015100	
C	4.78964000	2.84294500	-0.00021900	
C	3.44440400	3.22403100	-0.00024200	
C	5.76349300	3.84180600	-0.00025800	
C	3.05377500	4.56992300	-0.00030200	
C	5.38865500	5.17406800	-0.00032000	
H	6.81394400	3.57687500	-0.00024200	
C	4.04615800	5.54299000	-0.00034300	
H	6.14967000	5.94416300	-0.00035100	
H	3.76495400	6.58798600	-0.00039200	
C	1.65191300	-4.82092900	0.00007800	
C	0.44741300	-4.72619700	0.00006900	
C	-0.90432100	-4.54333200	0.00009500	
C	-2.08906500	-4.31378700	0.00012400	
C	1.65361000	4.82084300	-0.00032500	
C	0.44909100	4.72637000	-0.00033300	
C	-0.90262500	4.54337400	-0.00032800	
C	-2.08736200	4.31379600	-0.00030900	
C	-3.49611400	-4.13315900	0.00026300	
C	-4.32218700	-5.25860100	0.00040300	
C	-4.11642900	-2.87061800	0.00026200	
C	-5.70892900	-5.15746700	0.00054000	
H	-3.85169200	-6.23331100	0.00040300	
C	-5.52911000	-2.77328800	0.00040300	
C	-6.31793800	-3.91508600	0.00054200	
H	-6.30815900	-6.05891800	0.00064600	
H	-7.39822400	-3.83061700	0.00064900	
C	-3.49448200	4.13372800	-0.00014600	
C	-4.11529300	2.87143100	-0.00002200	
C	-4.32011200	5.25949500	-0.00011700	
C	-5.52801300	2.77465800	0.00012900	
C	-5.70689400	5.15890800	0.00003200	
H	-3.84923300	6.23401900	-0.00021400	
C	-6.31639100	3.91676600	0.00015600	
H	-6.30576900	6.06059400	0.00004900	
H	-7.39671000	3.83272300	0.00027300	
N	-3.58231600	-1.61510000	0.00014200	
N	-3.58167600	1.61570100	-0.00001900	
C	-4.61182200	-0.70102600	0.00020400	
C	-4.61154400	0.70203400	0.00013400	
H	-2.58953400	-1.33780600	0.00002300	
H	-2.58900400	1.33801200	-0.00011000	
C	-5.84311800	1.37254400	0.00023100	
C	-7.06934300	0.68609900	0.00039200	
H	-7.99982700	1.24048500	0.00046200	
C	-5.84366100	-1.37104900	0.00036600	
C	-7.06961500	-0.68411800	0.00045900	
H	-8.00031800	-1.23813500	0.00058400	

Cl	-0.88842100	-0.00024200	-0.00022600
Se	2.23320000	-1.79093500	-0.00004000
Se	2.23383200	1.79066200	-0.00018700

Cartesian coordinates of the structures of host-guest complexes **2** in the continuum DSMO model

Cl	0.79955400	-0.13147500	2.06228400
O	-2.16615600	0.03254100	2.93220100
H	-1.21782500	-0.01684300	2.68757500
H	-2.19101800	0.04829000	3.89203000
C	-1.92115000	-0.01520100	-2.18928400
H	-2.96072500	0.00774500	-2.49079000
C	-1.32559000	-1.24082500	-1.93741200
H	-1.90707800	-2.14764200	-2.02307100
C	-1.23954500	1.18256600	-2.04778900
H	-1.75469300	2.11691300	-2.21811100
C	0.00945300	-1.26495000	-1.54849700
C	0.09368700	1.14820300	-1.65363700
N	2.04197700	-0.09892500	-0.91773300
C	3.13580300	-0.07025600	-1.69923200
O	3.10534300	-0.06507500	-2.91958700
C	4.60489900	0.61085900	0.25275900
H	3.77552300	1.13579100	0.70884600
C	5.53540900	-0.68886900	-1.54719600
H	5.41031200	-1.18988800	-2.49749900
C	5.83574100	0.62852600	0.88440600
H	5.96958900	1.14672500	1.82251900
C	6.76781600	-0.69034200	-0.92310400
H	7.61456600	-1.19345700	-1.36607100
C	6.89533800	-0.02849600	0.28620600
O	0.71731800	-2.37804700	-1.24740500
O	0.87614600	2.23315400	-1.44609700
C	0.10275000	-3.64579900	-1.24767300
H	0.91203600	-4.36663000	-1.36538000
H	-0.58001400	-3.77737700	-2.08916100
C	0.32512100	3.52944600	-1.42517600
H	-0.40227200	3.68916300	-2.22348300
H	1.15935000	4.20799600	-1.60208100
C	-0.63377600	-4.01319100	0.03208400
C	-0.31164900	3.94491400	-0.10659100
O	-1.13827000	-5.12445600	0.11704200
O	-0.71163400	5.09466400	0.01058100
N	-0.66633600	-3.09403800	1.00389700
N	-0.38242300	3.02232500	0.86134000
C	-1.34777600	-3.30883200	2.26141800
H	-1.05343500	-2.49255500	2.91959100
H	-1.01284100	-4.24385200	2.71579200
C	-0.99980600	3.27964200	2.14407000
H	-0.57224800	4.17897900	2.59234200
H	-0.75193400	2.43484400	2.78515400
C	-2.86786300	-3.36444600	2.13262900
H	-3.16076000	-4.25008100	1.57458500
H	-3.29035800	-3.43760700	3.13872100
C	-2.51369600	3.46153800	2.06764600
H	-2.89418200	3.56690600	3.08736200
H	-2.75069200	4.36907600	1.51819200
N	-3.40865700	-2.21773000	1.44314900
N	-3.17117900	2.36419700	1.39983600
C	-4.22382200	-2.32707800	0.38742900
C	-3.97336200	2.53634800	0.34236900
O	-4.62775700	-3.38506700	-0.07885000
O	-4.26899800	3.62099200	-0.14325800

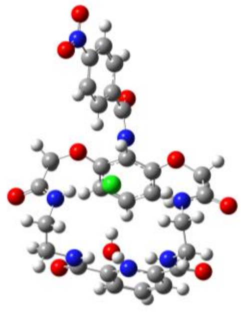

2

N	-4.18099300	0.10802800	0.29623700
C	-4.64568100	-1.01598700	-0.23424800
C	-4.52853400	1.26395900	-0.25478100
C	-5.48306900	-1.03311100	-1.34030000
H	-5.83022400	-1.97758200	-1.73352200
C	-5.36128800	1.34687600	-1.36153700
H	-5.61044400	2.31477200	-1.77181700
C	-5.84682000	0.17367500	-1.91068300
H	-6.49725800	0.19906400	-2.77562100
C	0.72099200	-0.07341700	-1.41533800
C	4.44754900	-0.05009300	-0.96133600
H	2.11792700	-0.17921500	0.09134900
H	-0.17726200	-2.21574400	0.89045200
H	0.00593000	2.10165200	0.71534200
H	-3.14780200	-1.29156900	1.76683100
H	-3.00411600	1.42287300	1.74121000
N	8.20229000	-0.01949400	0.95478300
O	9.12599800	-0.59040100	0.41077000
O	8.29550800	0.55792400	2.01911700

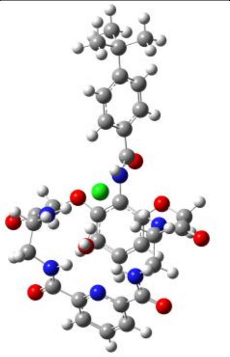

2b

Cl	0.52307300	-0.15530700	2.02414100
O	-2.41545900	0.01617700	2.97117200
H	-1.47402300	-0.04886900	2.70778500
H	-2.44754100	-0.22359200	3.90041700
C	-2.20909000	0.00870000	-2.17199800
H	-3.25379800	0.03072000	-2.45700100
C	-1.60659500	-1.21657100	-1.93469400
H	-2.18816800	-2.12468300	-2.01338400
C	-1.52698500	1.20653200	-2.03553600
H	-2.04628700	2.14174400	-2.19106900
C	-0.26485200	-1.23966500	-1.56818500
C	-0.18718600	1.17225900	-1.66318100
N	1.77911800	-0.07169700	-0.97356400
C	2.85921800	-0.07475300	-1.78200900
O	2.78376400	-0.09762500	-3.00188000
C	4.36724700	0.53478800	0.16657600
H	3.54011200	1.00919400	0.68227200
C	5.28688800	-0.60729500	-1.71913600
H	5.15943600	-1.05514100	-2.69710500
C	5.62080200	0.55012600	0.76325200
H	5.71948800	1.02386100	1.73072700
C	6.52921200	-0.59917600	-1.11555700
H	7.36270900	-1.05379300	-1.63799800
C	6.72778000	-0.01976200	0.14172500
O	0.44529200	-2.35362600	-1.27602700
O	0.59450900	2.26037400	-1.46450700
C	-0.16451000	-3.62212900	-1.29552400
H	0.64584000	-4.33992900	-1.42831000
H	-0.85118700	-3.74213400	-2.13643600
C	0.03695600	3.55143000	-1.41036100
H	-0.70314900	3.72440900	-2.19513500
H	0.86554300	4.23802900	-1.58680500
C	-0.89607800	-4.01084800	-0.01826400
C	-0.58341200	3.94373300	-0.07621900
O	-1.40167100	-5.12267500	0.05202300
O	-0.98478300	5.09059700	0.06333800
N	-0.92662700	-3.10401400	0.96507300
N	-0.63979500	3.00614900	0.87768900
C	-1.59835900	-3.33456600	2.22450400
H	-1.29272600	-2.53037900	2.89333100
H	-1.26433400	-4.27815700	2.66260500
C	-1.24155400	3.23953100	2.17274600
H	-0.80116900	4.12469200	2.63834000
H	-0.99367600	2.37711100	2.79055300
C	-3.11940400	-3.38151000	2.10590700

H	-3.42024100	-4.26033900	1.54030100	
H	-3.53806200	-3.46433700	3.11351500	
C	-2.75492400	3.43217400	2.12044700	
H	-3.11937600	3.52153400	3.14786200	
H	-2.99616700	4.35008300	1.58952800	
N	-3.65788000	-2.22524900	1.42991200	
N	-3.42895000	2.34975300	1.44501800	
C	-4.51344900	-2.32188600	0.40521400	
C	-4.23659000	2.53884000	0.39430200	
O	-4.95071400	-3.37363300	-0.04524000	
O	-4.52662800	3.62926200	-0.08065700	
N	-4.45705900	0.11271900	0.32931700	
C	-4.93433900	-1.00484200	-0.20365500	
C	-4.80433900	1.27478300	-0.20889400	
C	-5.78420800	-1.00900600	-1.30013100	
H	-6.14233400	-1.94899800	-1.69547800	
C	-5.64738600	1.37051100	-1.30662200	
H	-5.89483800	2.34331800	-1.70745400	
C	-6.14556400	0.20405900	-1.85884100	
H	-6.80467500	0.23941100	-2.71739900	
C	0.44885100	-0.04828700	-1.44235600	
C	4.18380900	-0.04790100	-1.08013100	
H	1.88136500	-0.15598600	0.03070000	
H	-0.43519300	-2.22467800	0.86127100	
H	-0.24918200	2.08893200	0.71420000	
H	-3.37903000	-1.30227100	1.74766700	
H	-3.28060900	1.40270100	1.77626800	
C	8.12126900	-0.02418500	0.76966800	
C	9.08627700	0.74886300	-0.14275700	
C	8.13743900	0.62818400	2.15272500	
C	8.61045200	-1.47297400	0.91362800	
H	9.15852000	0.29235800	-1.13122000	
H	8.75805800	1.78315600	-0.26861200	
H	10.08677000	0.76033900	0.29603600	
H	7.47921700	0.11043100	2.85365600	
H	9.15033400	0.58899700	2.55795000	
H	7.83687300	1.67718000	2.11130500	
H	9.60357300	-1.48707500	1.36840600	
H	7.93675800	-2.05082100	1.55063900	
H	8.67852100	-1.97616700	-0.05208000	

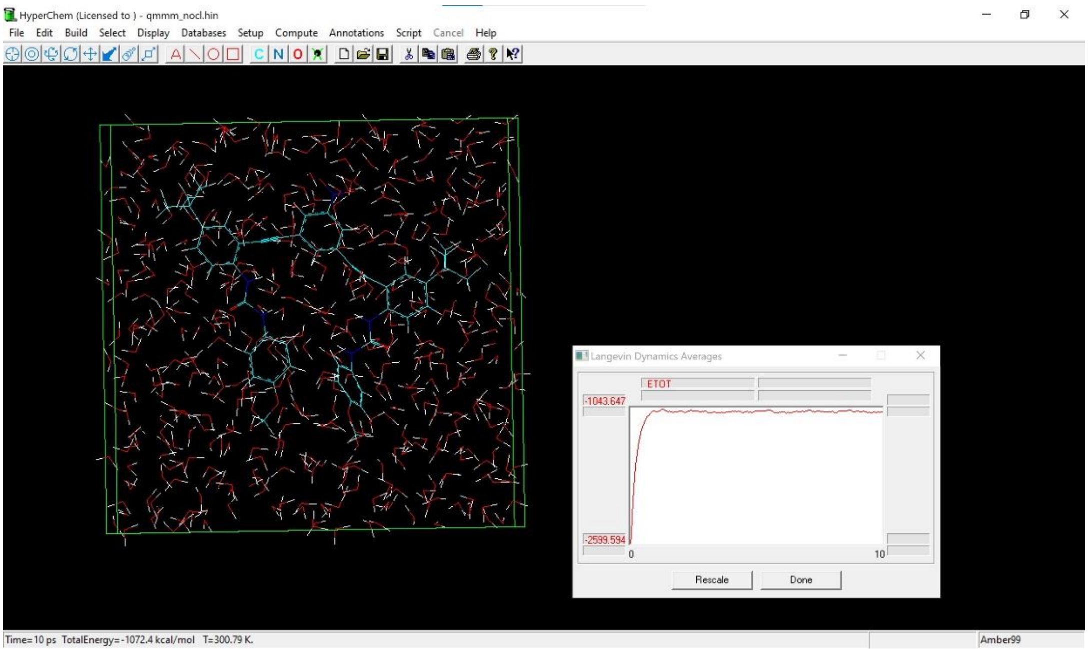

Figure S1. Illustration of the Langevin dynamics calculations

Cartesian coordinates of the QM/MM structures of host-guest complexes **1**

H 0.3155934918 -1.7026003591 -1.5540053652
C 6.6269169094 -3.6548154787 -1.0342701607
C 5.7724863525 -4.3839271296 -1.8527035418
C 4.4271699066 -4.0818023329 -1.9789778791
C 3.8651566787 -3.0196654775 -1.2707506173
C 4.7158816494 -2.2784404108 -0.4320934331
C 6.062793287 -2.6048712954 -0.3328912885
C 8.1193278138 -3.9262834556 -0.8896756599
C 8.5426784627 -5.1945464818 -1.622370708
C 8.8930368362 -2.7328450207 -1.4658797667
C 8.4582649326 -4.1052194627 0.5939190596
N 2.5392493642 -2.6269812892 -1.3571448393
C 1.5348010956 -3.2866844138 -2.0457676035
O 1.6847843092 -4.3918171337 -2.5372344517
N 0.3861556917 -2.5579251027 -2.1094357339
C -0.7912171882 -2.9572814559 -2.7559276331
C -1.9531049379 -2.2423780375 -2.4596130067
C -3.1509673611 -2.5804415002 -3.0471587973
C -3.2255677468 -3.6448338234 -3.9325421185
C -2.0762781245 -4.3436953724 -4.2536667732
C -0.8612689575 -3.9994164345 -3.674203516
O -4.4568090401 -3.9096624933 -4.4598507598
C -4.679398614 -5.2007467593 -4.9546355503
C 4.2526956543 -1.1828617727 0.3415610614
C 3.9701797539 -0.254804676 1.0496734702
C 3.8254897749 0.8130886662 1.9754892016
C 4.962901575 1.2004816872 2.684260431
C 2.6156516926 1.4672585141 2.1824737026
C 2.5399986378 2.4904986945 3.1274553168
C 3.6661643685 2.8653035888 3.8568772652
C 4.861992139 2.2247162545 3.5987235687

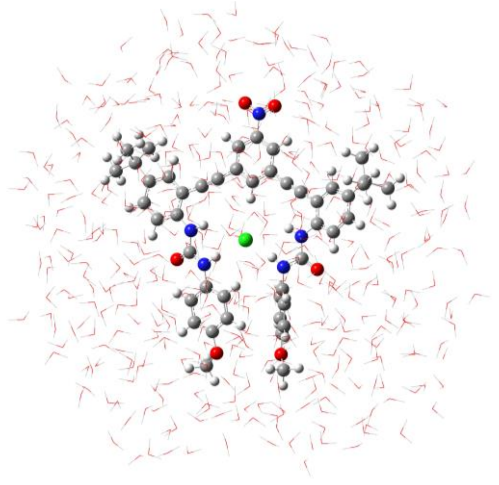

1

C 1.3263272665 3.2024282304 3.3289426732	
C 0.3420234966 3.8712609839 3.4961383232	
C -0.7993781746 4.6992600023 3.6541171124	
C -1.9272171304 4.5130498707 2.8412308643	
C -2.9954230269 5.3972966019 3.007240983	
C -2.9343086535 6.3963570363 3.9538690084	
C -1.835027022 6.5785944132 4.792908099	
C -0.7711996464 5.7170714904 4.6113888435	
C -1.8571862139 7.6672459568 5.8614668833	
C -0.541811724 7.7296875403 6.6387875294	
C -2.1107979337 9.0340316182 5.2143487352	
C -2.9921369604 7.3743172583 6.850978798	
N -1.9182564 3.4892386009 1.9042952018	
C -3.0436142253 2.9117052057 1.3361148584	
O -4.1726754523 3.2568471084 1.6291595669	
N -2.763539426 1.9279375373 0.4375550964	
C -3.8032458328 1.2166299297 -0.2010239284	
C -3.8388827828 -0.1687910398 -0.107340493	
C -4.8301965188 -0.8885418968 -0.7411555433	
C -5.795223568 -0.2352379131 -1.5000783379	
C -5.7851794342 1.1516424762 -1.5708348879	
C -4.7879944617 1.8681499658 -0.9242006968	
O -6.6681183934 -1.0348277081 -2.1785296708	
C -7.7540288358 -0.4200943295 -2.8235473665	
N 6.0759138932 2.7233249687 4.2594201769	
O 7.1313697799 2.1567185556 4.0644408327	
O 5.96778441 3.7127306934 4.9496664331	
H 6.1489776437 -5.2231055078 -2.4226250862	
H 3.8046691418 -4.672964789 -2.6266795345	
H 6.6707004464 -1.9925545411 0.3193589264	
H 9.6051719187 -5.3833402269 -1.45795124	
H 7.9878905834 -6.0623056945 -1.2599723555	
H 8.3831751412 -5.1129508116 -2.6960987197	
H 9.9688748328 -2.8981492597 -1.4036295603	
H 8.6264742825 -2.5783079342 -2.5129481394	
H 8.6597855271 -1.8132445728 -0.9283530962	
H 9.5107715231 -4.3705718476 0.7173816588	
H 8.2653439282 -3.1994072577 1.1666819535	
H 7.8481118748 -4.9023075777 1.0182945365	
H 2.2951269646 -1.7664596232 -0.88595063	
H -1.9066872059 -1.4100032943 -1.7652721696	
H -4.0505803213 -2.0312038271 -2.8179048662	
H -2.1049714378 -5.1647055286 -4.9575280512	
H 0.0258980709 -4.5540650308 -3.9289807961	
H -5.7580521974 -5.3072712449 -5.0656408942	
H -4.2049258856 -5.3703665157 -5.9285314729	
H -4.3185259752 -5.9681252891 -4.2611745136	
H 5.896395614 0.6924479414 2.5106731845	
H 1.7405454038 1.1690766001 1.6094172591	
H 3.6093023651 3.6595093523 4.586065417	
H -3.8774657021 5.2793551623 2.4016050632	
H -3.7853832464 7.0605939061 4.0419492119	
H 0.119874506 5.7975364303 5.2176301402	
H -0.6003148232 8.5148322263 7.3944188432	
H -0.3406982057 6.7918094572 7.1588555581	
H 0.3051280435 7.9532090508 5.9891168085	
H -2.1101182657 9.8147597458 5.9788495913	
H -1.3429881635 9.2660440338 4.4792314194	
H -3.0796247062 9.067771893 4.7131086605	
H -3.0096017178 8.1274669047 7.6421687819	
H -3.9654219105 7.3831289896 6.3580014222	
H -2.8623848515 6.3951908764 7.3090905835	
H -1.0315759777 3.0383415114 1.7386683229	
H -1.8316947971 1.5175306277 0.3620904449	

H -3.0695472659 -0.6755565252 0.4606475477<br>H -4.8544809426 -1.9664450669 -0.6669879974<br>H -6.5320045849 1.6896361934 -2.1383178775<br>H -4.781571063 2.9475308677 -0.9864621115<br>H -8.3767301368 -1.2199813599 -3.2217460192<br>H -8.3518874216 0.182763493 -2.1284501885<br>H -7.4316585348 0.2211603454 -3.6511773489<br>Cl 0.0576750917 0.1049453245 -0.0292036342<br>O 1.2949637813 -1.704875437 2.5603424739<br>H 1.1024735152 -1.3659105769 1.6759101805<br>H 0.4326476753 -2.0784846103 2.8375106251<br>O -2.8167545869 2.9787127436 -4.2332605915<br>H -2.5747379308 2.0305059493 -4.2225685413<br>H -2.4003831278 3.3155254295 -3.4219740504<br>O 1.850827025 1.8830514922 -2.1638089274<br>H 1.3428136546 1.3867687969 -1.5013425297<br>H 1.3076780164 1.7605279519 -2.9698192648<br>O -0.3081016815 5.6587825306 -0.0539140357<br>H -0.7363214363 5.1255202952 -0.7506048495<br>H 0.6319032939 5.5197545285 -0.210134712<br>O 3.5973985184 3.7619669195 -1.4146262599<br>H 3.0914779194 4.4584951134 -0.9690440681<br>H 2.9097903596 3.0875337836 -1.6206102051<br>O -3.2506495092 -2.9898927057 1.7403105309<br>H -3.2154370416 -3.8415600717 2.2195166417<br>H -3.7859887721 -2.4223203445 2.3422634687<br>O -2.0798723315 0.3674221633 -4.3295389431<br>H -3.0429192106 0.1726958872 -4.2799372186<br>H -1.7792654399 -0.3362505679 -4.9497580297<br>O -4.5629702959 -1.5257723451 3.479536992<br>H -4.7481546983 -0.5718932799 3.2537197046<br>H -4.032855009 -1.4499404972 4.2992495682<br>O -2.0646278875 1.0734702044 4.2132887146<br>H -1.1021863006 0.8994519999 4.2715092943<br>H -2.4420911623 0.2001142217 4.3636286463<br>O 0.333899361 1.5302694582 -4.4270485239<br>H -0.4901139392 1.038469698 -4.2036436743<br>H 0.0256087611 2.0383694694 -5.2130359854<br>O 0.5051001643 0.239717746 4.4777318493<br>H 1.2380799653 0.0636912528 5.1056515292<br>H 0.7710313898 -0.290132305 3.7117399157<br>O 6.6427991094 -0.3730288988 -2.6105204567<br>H 5.7266101751 -0.0516820333 -2.6970703556<br>H 6.8778378726 -0.1061529926 -1.7046585895<br>O 3.6268064471 -2.7468009666 3.3904916279<br>H 2.7909805113 -2.3049791524 3.1178392939<br>H 3.7116911816 -3.4407447281 2.7058373167<br>O 2.578521247 5.9723996988 0.2196181052<br>H 3.4174799534 5.6721288013 0.6176201231<br>H 2.0844306558 6.2474178539 1.0177708336<br>O -5.1942146807 0.9129372957 2.8740060169<br>H -4.8272289937 1.6521460771 2.3671858698<br>H -6.148407888 1.1543438402 2.9309151977<br>O -2.6189419146 -5.2634044729 -1.2025037487<br>H -2.4464306856 -4.3198855638 -1.3012170238<br>H -2.1413565006 -5.4668624599 -0.3505788674<br>O 4.5436513206 4.840566067 1.7556185857<br>H 4.0369204929 4.2736503191 2.3488876884<br>H 5.064310165 4.1950581348 1.2359739921<br>O -1.1383999345 -2.4456861076 3.4221006665<br>H -1.7349409294 -2.0130496706 4.0637892518<br>H -1.7348171245 -2.6350645902 2.6727683795<br>O -1.3355191458 -5.8482100245 0.9576766562<br>H -1.4949432334 -6.8110337446 1.1010408238	

H -0.3899042661 -5.7483287819 1.2570510776	
O 1.0744149534 -5.6721846377 1.9285043684	
H 1.976185125 -5.3437959665 1.7592538026	
H 1.1073003176 -5.8785355002 2.883950331	
O -5.0993504805 4.0269066537 -5.163870798	
H -4.2928966878 3.5634615293 -4.8431920034	
H -5.2371029638 3.6194562419 -6.0453532698	
O -5.6877615662 -4.1218020571 1.1359117041	
H -4.8354823854 -3.6580687718 1.1269857347	
H -6.3191098312 -3.4270106435 0.8693965553	
O 0.6812925146 -2.9780595998 -6.9350806554	
H 1.1358212846 -2.7120817603 -7.7520035663	
H 1.4281264215 -3.0465778533 -6.3014535472	
O 5.819979164 3.1336140958 0.0904213025	
H 5.1361231765 3.3239805147 -0.5812597772	
H 6.5992952636 3.6363164682 -0.2253082962	
O -1.6083454639 4.540509081 -2.2094781948	
H -2.4793430621 4.9633367645 -2.0825430756	
H -1.2488247376 4.9952670854 -2.9978936064	
O 1.6393661787 6.7462764961 2.6758059834	
H 2.0745680517 7.0665415248 3.492514161	
H 0.8610796717 6.2879115554 3.0148876003	
O -0.1010578337 2.2076611775 6.6451546526	
H 0.4355179032 2.0871739651 5.8497642529	
H 0.4484715088 2.8253593441 7.1936699931	
O 2.5895080148 -0.3007622395 6.0566544607	
H 3.4687195653 -0.4093375379 5.6269149902	
H 2.80964332 0.2320134876 6.8501334995	
O -6.4770006177 4.783709768 -2.9500967075	
H -7.3141537006 4.3102718577 -2.8058840572	
H -6.1239773149 4.3724619821 -3.7654494987	
O -2.1490071892 -7.3912275608 -2.8326650047	
H -1.2430853224 -7.7045430307 -2.5966698537	
H -2.2060303123 -6.5298546688 -2.3630800263	
O -2.6878147486 -1.4709590082 5.4687141807	
H -2.3348534028 -0.7793731127 6.077994229	
H -2.6657618544 -2.2519775532 6.0507682785	
O -2.9559863917 -5.3372899013 3.0190978189	
H -2.429349817 -5.5018198605 2.2055847274	
H -2.4421472034 -5.8497805102 3.6861635532	
O -1.3902623266 -1.4498370573 -6.1468933488	
H -0.638440262 -2.041413811 -6.3727335788	
H -2.1686001681 -2.0026213571 -6.3803375075	
O -4.7186254475 -0.0931025537 -4.4826816909	
H -5.2514407388 0.1524293052 -5.2629667545	
H -5.3783671845 -0.4394314138 -3.8709891887	
O 4.3125739749 -4.024097238 5.6427747019	
H 3.9773960186 -3.4791496421 4.8940898118	
H 5.0272518626 -3.4676189406 6.0082614703	
O 2.3085632594 -0.4960123216 -4.6903683231	
H 2.5114477919 -0.4996375694 -5.6567079209	
H 1.5462708034 0.1075677167 -4.6379974655	
O 4.1681870525 0.6690079619 -3.1732562253	
H 3.5436134007 1.1757093078 -2.6290321303	
H 3.5540979459 0.1082450369 -3.6988345651	
O -9.600986939 -1.1549789773 3.8446822091	
H -9.4080733173 -0.2855682078 4.2448389104	
H -8.6970357332 -1.5294442207 3.7289811947	
O 2.8164230802 -3.1774555431 -5.3114128007	
H 2.6637635528 -2.3496161278 -4.8203943578	
H 3.7982160437 -3.2076477524 -5.3727993157	
O -7.1514945195 -2.1212762629 3.6123686836	
H -7.2041532385 -3.1085471627 3.6588453799	
H -6.184951696 -1.9630871936 3.503972957	

O -1.9496245147 7.6513429744 0.7338291282<br>H -1.4279355108 8.2491846718 1.3034252229<br>H -1.3123390047 6.9332488763 0.525591303<br>O 5.2886631779 -5.8334990015 3.873053816<br>H 4.9192429417 -5.2737017286 4.5817765292<br>H 6.2198657855 -5.5187901573 3.830651127<br>O 7.2252144281 0.8072795509 -0.1678301339<br>H 7.6801781763 0.6695733822 0.6851937293<br>H 6.6035346961 1.5331989382 0.0386961742<br>O 1.8662470261 9.2068307995 1.4582894793<br>H 2.8283908889 9.4410945527 1.4625169766<br>H 1.8643533957 8.3119512002 1.843664677<br>O -4.1010840236 5.7235252372 -2.0516879649<br>H -4.980418956 5.3103213514 -2.1761681336<br>H -3.9874898943 6.1868738388 -2.9043324152<br>O -2.4504852173 3.4614654601 7.1684320525<br>H -3.0194789602 3.347860733 6.3775979373<br>H -1.6045183759 3.0730797052 6.8695062502<br>O 7.8082205591 -5.0325643085 3.9686352313<br>H 8.5287011611 -4.3911839132 3.7989330976<br>H 7.9633679861 -5.298126459 4.8966557317<br>O -0.6657788516 9.7533984255 1.9428836423<br>H -0.729142175 10.7183955656 1.7667720213<br>H 0.2768064071 9.5668022144 1.7323526324<br>O -8.5606891421 0.3752271288 0.560705134<br>H -8.1294853477 -0.4941466905 0.4773202094<br>H -8.1190452998 0.7630567328 1.3334629074<br>O -3.5655131289 -2.8649913026 -6.8565460543<br>H -4.3858316785 -2.4319278881 -7.1891573828<br>H -3.8876174778 -3.2856543906 -6.0357855921<br>O -6.0736963344 -2.0805389032 6.2247896958<br>H -6.1211913382 -1.1966371579 6.6402247282<br>H -6.585438177 -1.963722474 5.4101514236<br>O 4.3841966008 9.9113104313 1.3355246181<br>H 4.5157227971 10.8828379148 1.3789133256<br>H 5.0461409202 9.634401587 0.6650867621<br>O 1.1765706674 3.913139522 8.1908346664<br>H 1.9928562906 3.9745387031 8.7168268928<br>H 0.5761888781 4.5117022435 8.6725340314<br>O -3.8446845207 2.9479984569 4.8953770132<br>H -3.2399738558 2.2285745667 4.5924071932<br>H -3.960473945 3.465557196 4.0888557734<br>O 3.7524326503 -4.9876233553 1.802316981<br>H 3.963147932 -5.6328678748 1.0952302439<br>H 4.2876178763 -5.3334331647 2.5423797312<br>O -3.8311032369 -8.2514004363 -0.8583728536<br>H -3.094877498 -8.1073223271 -1.477467825<br>H -4.3962413478 -7.4749607193 -1.0352838473<br>O -6.361557059 0.3467546673 7.3867944187<br>H -5.4505384001 0.3792079314 7.7737150222<br>H -6.3869160783 1.2175000883 6.9117005749<br>O 4.9478455484 -0.9427532862 4.9856655116<br>H 4.6357222302 -1.5202690422 4.2651041575<br>H 5.6197828298 -1.4802474753 5.4340268459<br>O 4.1170361865 4.4447894342 -3.9895209204<br>H 4.0996945802 4.1158201629 -3.0682123595<br>H 3.1625253146 4.3752612199 -4.2339132999<br>O -2.0709702179 -8.3522940633 1.3196737089<br>H -2.4093850152 -8.7060854684 2.1711050795<br>H -2.8153894376 -8.5489508556 0.7117858978<br>O -7.7507710242 1.5656113818 2.9960991248<br>H -8.3076113504 2.2793732949 2.6083201019<br>H -8.1692406063 1.4448563946 3.8709363856<br>O -0.8380635561 3.1293998672 -6.2456282813	

H -1.6429376133 3.3144049456 -5.7369531524<br>H -1.007259385 3.580151962 -7.0960122587<br>O -4.7518532089 9.7332349487 -2.4658541669<br>H -3.9809013497 9.7992118729 -1.8651923406<br>H -5.3968927494 9.2411553708 -1.9128562507<br>O 0.2212646727 -8.4199382793 -2.1503745421<br>H 0.2988220835 -8.5926669847 -1.1827152115<br>H 0.2730042958 -9.3239981436 -2.5133898782<br>O 0.1470031462 -3.6612070175 5.4661110812<br>H -0.2589019448 -3.2592008805 4.6685763103<br>H -0.583797129 -3.6116116187 6.1200094628<br>O -8.6145107002 8.8243515447 -4.0451823733<br>H -8.7411948917 8.6930292886 -3.0812893566<br>H -8.38867175 9.7782415444 -4.092259844<br>O -1.3910249767 0.4709559141 -8.0164920169<br>H -0.4915161502 0.8719127551 -7.9409812395<br>H -1.3988407802 -0.1459147639 -7.2537459276<br>O -2.7937668684 -0.9919161774 -9.7740682605<br>H -2.2202458053 -0.4950742137 -9.1529553493<br>H -2.6529053499 -1.9174617215 -9.5107821707<br>O -4.0517759256 6.5061882731 -4.6574769113<br>H -4.5668282911 5.7041021526 -4.8868752573<br>H -3.2907215212 6.437920306 -5.2758475156<br>O -6.6140009284 -3.8125481747 -1.8955766197<br>H -6.5584397322 -2.8514632383 -2.0229300299<br>H -7.5730872451 -3.9566703244 -1.8216332662<br>O -5.2656818459 3.0001262085 -7.6688095578<br>H -4.4824770651 2.6771445386 -8.1710589124<br>H -5.9165466214 3.1828249978 -8.3873779441<br>O -7.2537888575 6.7572745241 -4.9492947471<br>H -6.8518486919 6.4293086214 -4.1290094326<br>H -7.7460304625 7.5592438858 -4.6429574022<br>O -5.1642010748 -6.0007452261 -1.666049581<br>H -4.2495539587 -5.6491888917 -1.5714288799<br>H -5.6770782499 -5.1588950295 -1.7972019015<br>O 8.8127216949 2.0426437264 -2.0017356024<br>H 9.5183412315 1.3676743539 -2.1278861415<br>H 8.2116656279 1.5700487612 -1.3913770061<br>O 2.6063870156 -8.7857459376 1.6472663893<br>H 3.2068434151 -8.1312059723 1.2397781853<br>H 1.811468812 -8.7295370612 1.0765789542<br>O -0.5343454167 5.727501133 -4.4227779352<br>H -0.1902003447 6.6244058065 -4.202442573<br>H -1.0856203597 5.909901606 -5.2190556146<br>O -1.3812075843 2.2662304971 9.7343664402<br>H -1.8893870239 1.5492841434 10.1921026915<br>H -1.9579847052 2.5053990459 8.990866885<br>O -6.1681736102 2.7145864587 6.2512752535<br>H -5.3528542453 2.8057207432 5.7069408981<br>H -6.8238450582 3.2415726166 5.7449568339<br>O 1.0727730889 -6.0087716948 4.621090378<br>H 0.8032099566 -5.125904448 4.9586873448<br>H 1.8347964374 -6.2240202152 5.196745416<br>O -5.1090003506 -5.5497215852 4.5617443896<br>H -5.9103665321 -5.1664534579 4.1339189914<br>H -4.3997316133 -5.3931543952 3.8930425942<br>O 6.4243263912 6.3468575808 3.0634421473<br>H 6.0108116266 7.1744551369 3.3849077215<br>H 5.6573789047 5.8463928909 2.7258690329<br>O -9.4572683341 3.3673635711 2.0699242086<br>H -9.5294740231 4.207064453 2.5686603263<br>H -10.2058437273 3.4224922266 1.4578777705<br>O 2.0834072669 -2.067257367 -9.2654740929<br>H 1.1882331375 -2.2573468409 -9.6233493446	

H 2.6261669369 -2.1367661695 -10.0699869095<br>O 6.5427029827 -2.6366937737 6.5121882844<br>H 6.6649595981 -3.3593308723 7.1723211194<br>H 7.4683279295 -2.4918193268 6.1915514912<br>O 5.7772944959 1.3210807415 -5.2839352782<br>H 6.5315877225 1.8351216786 -4.927240636<br>H 5.201581535 1.211977189 -4.4986964677<br>O 1.6910023092 -2.4977442793 7.3091773889<br>H 2.0807504169 -1.7317026497 6.8317391898<br>H 1.2197215046 -2.9633093167 6.5813890056<br>O -0.0977957372 -5.6319860865 -6.8192088618<br>H -0.0866937337 -4.6639216466 -6.9341059967<br>H 0.8498708433 -5.8167974765 -6.666232174<br>O -1.6392877927 0.1651197012 7.2732932886<br>H -1.0628309934 -0.3297196395 7.8917608325<br>H -1.0546088731 0.920807395 7.0042440245<br>O -9.1900170736 1.1080786104 5.2704330686<br>H -10.019941347 1.57584978 5.4629331687<br>H -9.0563534159 0.5652484298 6.0738256604<br>O 3.1897285629 -6.3135565621 6.2699336058<br>H 3.5836265854 -5.4207376787 6.1080525873<br>H 3.446973592 -6.4798011876 7.1958306829<br>O 10.2846347451 -1.288486051 -4.581191151<br>H 9.8445663553 -2.1532088311 -4.7455070123<br>H 10.1949196037 -0.8364332833 -5.4376643049<br>O 0.2520206968 -9.1317064448 0.4072193265<br>H -0.6037394207 -8.8104947351 0.7877037491<br>H 0.2372851569 -10.0730494256 0.6550975145<br>O 6.9259356263 -1.1642851957 -5.1810784446<br>H 6.5223955173 -0.3059653626 -5.4189630305<br>H 7.0355840638 -1.0501972362 -4.2110835312<br>O 9.8609320243 -3.2803414049 3.5125544304<br>H 10.744925856 -3.668700662 3.6586755259<br>H 10.0141432124 -2.7513668069 2.7028590126<br>O 1.5631210773 4.1085963255 -4.5951270642<br>H 0.7853176847 4.7199781258 -4.5525906207<br>H 1.1504626633 3.2538614597 -4.3868669098<br>O 2.8962576241 -0.4867707066 -7.2629040229<br>H 2.7087749548 -1.1135175782 -7.9999252955<br>H 3.7459492195 -0.0727398392 -7.5458398351<br>O 2.1670265275 7.911746968 -1.6964094724<br>H 2.2566328803 7.205887456 -1.0233783766<br>H 3.1083378493 8.1397330953 -1.8778163052<br>O 0.8859089037 0.9612183664 9.8336999425<br>H 0.0783481867 1.5280485729 9.7443641836<br>H 1.3311277817 1.3853229982 10.5965902206<br>O 2.3491083301 -7.1467191671 -3.2296478313<br>H 1.5435459019 -7.5601826389 -2.8529752408<br>H 2.1909317541 -6.2031821612 -3.0704071947<br>O -7.5070751792 -4.7064874338 3.7665508339<br>H -8.2232636681 -4.8235638108 4.4279433185<br>H -7.8723700654 -5.2160625713 3.0029695084<br>O -4.2303537807 5.600878473 -8.0710787308<br>H -5.0037985848 6.1877750723 -7.9000565588<br>H -4.5957702929 4.7217040295 -7.8859693041<br>O -1.033330703 10.0705740672 -2.7850805722<br>H -1.6234869675 10.546927793 -3.3949944562<br>H -1.5716121171 10.0319983893 -1.9647111176<br>O -4.4708292925 6.8414527347 0.4041585339<br>H -3.5544788058 7.1245157226 0.6213920272<br>H -4.3233901333 6.2943104247 -0.3946303941<br>O -2.7264597819 9.8346312801 -0.6770889624<br>H -2.9728183284 10.3697157308 0.0984865564<br>H -2.4628290584 8.989881282 -0.2576160266	

O -4.3282679738 -8.707139492 -3.8965642728<br>H -3.5434169725 -8.2298309298 -3.5586571802<br>H -4.6715741583 -9.158833717 -3.1099410276<br>O -4.2103886585 9.1644973862 -4.9608018966<br>H -4.16439463 8.1838247631 -4.8756008878<br>H -4.499993154 9.4165203434 -4.0465625164<br>O -0.3451602629 5.7002595727 9.7007345451<br>H -1.2736356976 5.4039378147 9.6031459299<br>H -0.3199789623 6.0268167829 10.6116302222<br>O -8.8448089609 -0.6067162415 7.3496117575<br>H -8.7970027286 -1.5896359623 7.3049580968<br>H -7.899824519 -0.3481478427 7.4417929181<br>O -3.9744297647 0.3732914859 8.4536239195<br>H -3.1116772949 0.3694333077 7.9691553915<br>H -4.0439914292 -0.5765873395 8.7179475036<br>O -7.651151388 -2.2068966274 0.9013989237<br>H -7.5479521064 -2.0512019445 1.8549731606<br>H -8.4453580992 -2.7878814891 0.8626537324<br>O -6.1708671222 -6.6838882321 0.7770936742<br>H -5.9376293532 -5.7620340227 1.0454639214<br>H -5.9656741751 -6.6475695985 -0.1745367519<br>O 3.1155373596 0.8550812826 8.4101894096<br>H 3.5173921586 0.2173060942 9.0351002089<br>H 2.2426542377 1.0206698562 8.8326780962<br>O -0.8447978232 -7.8266048429 -5.2909820061<br>H -1.4330994572 -7.6135590422 -4.5514957238<br>H -0.716585066 -6.9734491511 -5.7379077985<br>O 8.1230746546 4.3484276638 -0.6772871864<br>H 8.5628450768 3.5875182389 -1.1062210255<br>H 8.2771128222 5.0705041227 -1.3195206654<br>O 6.4035728015 8.9936062427 -0.2314061001<br>H 6.7959787289 8.114571646 0.00747391<br>H 7.0007064055 9.6047944895 0.2435576916<br>O -2.4284490962 -3.6617747916 -9.1108118734<br>H -2.7880086965 -3.4825342626 -8.2117986189<br>H -3.100735575 -4.273958447 -9.4771263561<br>O -4.3994456314 2.5880134039 9.8204807862<br>H -4.7140025618 2.2867281713 10.6930005541<br>H -4.2538146681 1.7322892207 9.3414080728<br>O -1.4981289935 -6.8294131856 4.6768177672<br>H -0.538223039 -6.6693941869 4.5395462794<br>H -1.5402432869 -6.9147908757 5.6549112945<br>O -8.754310939 8.2137438004 -1.4438091038<br>H -7.7940335962 8.238056056 -1.2194113497<br>H -9.1310765094 8.7341992504 -0.698362402<br>O 5.4667531614 -3.3715137786 -5.6049454659<br>H 6.0583168434 -4.0871338882 -5.2919400975<br>H 5.996300601 -2.5658858841 -5.4142616262<br>O -1.8402794248 -11.2607975283 -0.820128334<br>H -2.427832613 -11.6241358527 -1.5149676786<br>H -2.5047439348 -10.9802589488 -0.1384543043<br>O 0.4877516408 8.073835314 -3.7390368946<br>H -0.1337705026 8.7670866487 -3.4214067767<br>H 1.0772298862 7.9585315213 -2.9550784195<br>O 5.1905239476 8.7189991111 3.5830836668<br>H 5.9110804601 9.3223580202 3.8679283143<br>H 4.8928771019 9.1420216581 2.7492966569<br>O -6.3225554838 7.1174930573 -7.4310271373<br>H -6.6578391826 6.9676752296 -6.5179084136<br>H -6.1738478477 8.0848862018 -7.4375381127<br>O 10.6021549905 0.079008819 -2.3368407041<br>H 10.4105682304 -0.4761640952 -3.1320089227<br>H 11.1720495695 -0.5019397074 -1.8039175821<br>O 1.1168181181 10.2112790627 4.2937709611	

H 1.3534189544 10.9245018955 3.6720255431<br>H 0.3154762084 9.8421543808 3.8964528329<br>O 4.7451453462 12.6064970169 1.5526339015<br>H 4.0366406058 13.1557019466 1.1689931063<br>H 5.4585068709 12.694360963 0.8926431047<br>O 5.2273344536 -8.4900959026 4.1926597644<br>H 5.1524483343 -7.5114331456 4.1291666507<br>H 4.451919806 -8.7249615161 4.7365349766<br>O 10.7266769575 -1.78982973 1.3716844487<br>H 11.1806938628 -2.4096432764 0.7811787513<br>H 10.8032569925 -0.9454825273 0.9096164557<br>O -5.5082834605 -9.0174245664 1.958009681<br>H -5.702610884 -8.1620596943 1.5121731935<br>H -5.6249105314 -8.7752139445 2.9057220622<br>O 5.6738200732 6.5119889493 -3.4168766794<br>H 5.0782060014 5.7619782124 -3.6503656903<br>H 6.0712837057 6.7425147339 -4.2821230655<br>O -3.6647719055 -10.7219147582 0.9880274448<br>H -3.7553421528 -11.5672875539 1.4596625182<br>H -4.3389103588 -10.1495563572 1.4201695665<br>O -13.8346198875 -1.4944391017 5.8004222283<br>H -12.8794397666 -1.6801308947 5.8945265269<br>H -13.8425917364 -0.6741463168 5.2844885302<br>O -1.6492859781 -3.2214240365 7.4251791541<br>H -1.9443121735 -3.85630383 8.1072803691<br>H -1.2454753441 -2.521318849 7.9648153979<br>O -0.2194595262 -2.9127155107 -10.2962930279<br>H -1.0256184995 -3.2408954417 -9.8252497268<br>H 0.1706382126 -3.7419951214 -10.6352764519<br>O 2.6178467733 -5.6295784629 -6.4648249504<br>H 2.6546241665 -4.7752538566 -5.9881285019<br>H 3.4851990755 -6.0241917777 -6.2374495683<br>O 6.7915771332 -4.5758461491 8.3164747924<br>H 7.2265236312 -5.2024755937 7.6971309894<br>H 7.1859295756 -4.8300000496 9.175632663<br>O -10.241993614 6.191743872 -2.2469648664<br>H -9.6360847925 6.9318595912 -2.003844904<br>H -10.6020125551 6.504993167 -3.1090836822<br>O 7.9529348664 -6.2044107601 6.4823393838<br>H 8.7188300225 -6.6939464821 6.1381485891<br>H 7.3623193913 -6.9294196647 6.7979444184<br>O -0.9262256138 12.3899307654 1.6080502571<br>H -1.2279628871 12.7569641839 0.737044878<br>H -1.6253859263 12.7129502476 2.2145265134<br>O 2.2070646865 6.9183018109 9.7015244459<br>H 1.2980507494 6.6546043742 9.4779612784<br>H 2.2845243969 7.8032256638 9.3056940578<br>O 7.4105163943 6.7229183809 0.5948937871<br>H 7.1556139369 6.5557946473 1.524376533<br>H 7.714563307 5.8504680885 0.2894545407<br>O -3.0309324428 2.3083377075 -9.004158274<br>H -2.422649887 3.0806553504 -8.9413311328<br>H -2.480242337 1.5981168586 -8.5946407828<br>O 1.7572944541 10.49701356 -0.9044003716<br>H 1.6689209507 10.2251697822 0.0293739345<br>H 1.7830617884 9.6249925673 -1.3444790426<br>O 8.4899360897 0.6827114561 2.2817727391<br>H 8.009920773 1.2390036995 2.9328034458<br>H 8.727541475 -0.0917996628 2.8034896025<br>O 10.4484787477 0.147929696 -7.0076364797<br>H 9.9819735934 0.5928179625 -7.7373541081<br>H 10.7436290599 -0.6895227221 -7.4204058924<br>O -11.1852522501 -1.9791179801 5.9458918325<br>H -10.6790371166 -1.7670011395 5.1403595405	

H -10.550265633 -2.5015633962 6.4606658353<br>O 4.6697065229 -3.4854789322 9.667263986<br>H 3.843348086 -3.9464225422 9.3821475572<br>H 5.3520610835 -3.9164617871 9.1102578314<br>O 2.8627880343 8.0813244327 4.7087168233<br>H 2.3129833484 8.888524328 4.6700972379<br>H 3.7105208277 8.3693664689 4.3000389766<br>O 8.4077682045 4.4823850467 3.2178320046<br>H 7.8040895858 5.249962521 3.2937437208<br>H 7.8258775207 3.7551646728 3.5033830536<br>O 4.4215812776 -7.1663059951 0.3893592429<br>H 5.191976355 -7.5966973284 0.8148737598<br>H 4.3961071047 -7.6094962627 -0.4862259469<br>O -4.5923669993 0.7544019818 -10.645134077<br>H -4.1599324844 1.4615699708 -10.1315545838<br>H -4.0499472915 -0.0263042387 -10.384113766<br>O 7.0915073826 -0.3651604887 -8.9846468827<br>H 6.6475125321 -0.9369737187 -9.6519767314<br>H 6.3200501845 0.0857192701 -8.5766183224<br>O 5.0507658836 0.941367503 -7.8678320993<br>H 5.0550985186 1.7489658947 -8.4076855746<br>H 5.3329012169 1.2566669221 -6.9787036326<br>O -11.1223709899 6.300565934 0.2722326652<br>H -10.8590909471 6.2539274016 -0.6745872063<br>H -10.315414674 6.5925008702 0.7088548294<br>O -0.1126513743 -1.3420112117 8.941276056<br>H 0.6364268158 -1.7686339967 8.4664278992<br>H 0.3241027784 -0.5577753806 9.3594006066<br>O -1.6178475666 3.526554852 12.1610609814<br>H -1.5487598823 3.1912483848 11.2439028683<br>H -2.5235264235 3.8685724791 12.2039176129<br>O -6.3135530766 0.5876856113 -6.575857895<br>H -7.2865637765 0.635760581 -6.4946176972<br>H -6.0725712624 1.4595206313 -6.9374291641<br>O -11.8417658857 -0.8063681303 2.4741177345<br>H -10.9765589636 -0.9635178794 2.9184947769<br>H -11.5929191403 -0.2244322258 1.7252780194<br>O -13.1938896983 0.7321384103 4.1760503783<br>H -13.5137019374 1.4154188096 3.5608433438<br>H -12.7697758496 0.089796042 3.5627401601<br>O -5.0510393911 -4.3992071389 6.9325528939<br>H -5.0084019078 -4.8393378347 6.0479037156<br>H -5.3910427854 -3.5031600493 6.6807592465<br>O 6.2874066551 -8.0212896461 7.5273649721<br>H 5.4951857673 -7.79203462 8.0397055793<br>H 6.2685723101 -8.9929436699 7.5025877363<br>O 0.1703455349 -11.8887991098 0.8770117858<br>H 0.8763300524 -12.2078667844 0.2703769795<br>H -0.6010267071 -11.8078272413 0.2763093324<br>O -2.6666488 -5.2225490829 -12.6147935008<br>H -2.9254867632 -5.3671829374 -13.5266527807<br>H -2.9565545114 -4.2961899995 -12.4528309497<br>O 0.6926616768 6.8382733146 12.1715219166<br>H 1.4811651803 6.883225435 11.6149524225<br>H 0.8093531549 5.9974702301 12.6558517676<br>O -7.2396000511 -7.2982019431 -2.9221774687<br>H -6.4324132331 -6.8619752588 -2.5869446978<br>H -7.0467624408 -7.4066032747 -3.8752874175<br>O -2.7380589709 -9.0874596963 3.8637995336<br>H -2.4067232985 -8.2119239995 4.157539704<br>H -2.0449562124 -9.687728474 4.2166769333<br>O 8.4475676481 -7.1161453461 2.268577047<br>H 8.0750837585 -6.3584030232 2.7542969863<br>H 7.6520485995 -7.6385050393 2.0284749904	

O 3.2999580171 4.423087465 9.8967723886<br>H 4.2299382388 4.4532250797 9.5904721491<br>H 3.037014343 5.3661207124 9.8845779096<br>O 4.4643716783 -0.8886984275 9.977012697<br>H 5.1850277233 -0.7265797107 9.3284421079<br>H 4.4305901469 -1.8792127801 9.9568568224<br>O -8.5702277624 -6.1699757078 1.8503231312<br>H -7.7660823358 -6.4598349433 1.3659571897<br>H -9.008205632 -7.030427756 2.0445981334<br>O -11.1624131571 0.8861457311 0.4855507286<br>H -10.1890686989 0.7283409165 0.4146706569<br>H -11.2127176902 1.8557682004 0.5237320985<br>O -2.9018569508 4.7974998415 9.3765497692<br>H -3.4773446047 4.0792556298 9.7119262023<br>H -2.7144799148 4.45893701 8.4667569141<br>O 1.1060212521 1.3536813494 -7.8913532938<br>H 1.5237902803 2.1892136963 -7.5828350856<br>H 1.7447503357 0.6770804724 -7.5732099153<br>O 8.9852137707 -2.059019835 5.7139908253<br>H 9.2946245455 -1.1436339146 5.5387586228<br>H 9.285690493 -2.5347856294 4.9094827231<br>O -13.5703494733 2.8625586091 2.3910026704<br>H -12.8356404677 3.1530846979 1.8181023362<br>H -13.4472747835 3.4367162112 3.1820777875<br>O -10.8291223413 7.1657136305 -4.6670954134<br>H -10.1914298923 7.8947987626 -4.7143204682<br>H -10.8847862922 6.8562131922 -5.5902971127<br>O -11.198459497 0.2978904771 8.1375081467<br>H -10.3200378583 -0.0781216235 7.8835317871<br>H -11.7746243994 -0.4728119362 8.0730592311<br>O 10.6530062221 2.1569774081 -5.2590947257<br>H 9.7084803616 2.2489035815 -5.0519999203<br>H 10.6583456606 1.400773051 -5.8876628716<br>O -2.1219622828 6.4111545898 -6.473710978<br>H -2.7009870522 6.0598098912 -7.1815416336<br>H -1.9015473328 7.3094724674 -6.815734946<br>O 0.8487429105 4.2834940711 13.06116117<br>H -0.0591817996 4.0569957018 12.7906401175<br>H 1.3826946663 3.6059457072 12.6139993801<br>O 2.3531632003 2.5231789957 11.5316491588<br>H 3.1271769036 2.1094882011 11.9248517685<br>H 2.7348767142 3.2151973632 10.9461516307<br>O 7.6897157403 -9.3472698043 4.9475383764<br>H 7.4152539795 -9.8470145331 5.7378943526<br>H 6.8307049175 -8.9961162851 4.6343976968<br>O 12.0025785911 -5.5192642715 1.6901937128<br>H 12.1496049861 -4.9222098965 0.9303279926<br>H 12.2274097657 -4.9447862935 2.443614567<br>O -3.3436286282 -10.9520345309 -5.1852442854<br>H -2.9452274864 -10.6789630887 -6.0306127646<br>H -3.7163930519 -10.1203125282 -4.8540188815<br>O 4.6726657495 8.6223533193 -2.2035150036<br>H 5.0844569982 7.8337122237 -2.6302263202<br>H 5.3308652571 8.835164433 -1.4974888513<br>O 3.1583851752 -2.2925437007 -11.8402900838<br>H 3.3007509557 -3.2145051343 -12.1342913139<br>H 2.4673765759 -1.9879320328 -12.4669202056<br>O -3.315657931 13.1618932947 -2.9020461886<br>H -3.0189674092 12.4467695321 -3.5006072951<br>H -3.7481950115 13.7671480394 -3.5209868727<br>O 3.7486033155 12.2190009587 4.20001206<br>H 4.4090600908 12.3786734596 3.5088432248<br>H 2.9151924998 12.3379204146 3.7124352982<br>O 12.3080927463 1.9594246508 -3.1115924758	

H 11.8781750231 2.2519233911 -3.9390771536<br>H 11.6691686034 1.2878248706 -2.7926938163<br>O -1.8419261238 -7.1550275348 -8.2606161074<br>H -1.6233373377 -6.9467865936 -9.1964739329<br>H -1.2022078618 -6.5962511427 -7.7700808988<br>O 9.720807156 -7.5778396729 4.6948343519<br>H 9.1269486263 -8.327309677 4.8906954846<br>H 9.454887736 -7.3459463144 3.7901634434<br>O -3.602135462 -12.1135257835 -2.7164969219<br>H -4.3726894581 -11.5769511116 -2.4555050128<br>H -3.4799490904 -11.867954979 -3.6576466209<br>O 1.2261175921 -9.3582717516 3.8642197454<br>H 1.7032355751 -9.0776572708 3.0553287447<br>H 1.8797564252 -9.1637822728 4.5601731337<br>O 2.7337776847 -11.4923737943 2.188826763<br>H 1.8171579695 -11.6998053493 1.9662055512<br>H 2.8025648559 -10.5453257378 1.9658190445<br>O 11.5091170283 5.4881720817 -1.9973108889<br>H 11.2922790599 6.0913747875 -1.2691549209<br>H 11.7666282464 4.6764934328 -1.5331758558<br>O -9.0344494241 -4.3643736308 -4.5984361983<br>H -9.6451474954 -5.128700087 -4.530384361<br>H -8.9004150986 -4.1356536928 -3.6706953864<br>O -6.2984055498 4.0385345235 8.6155254091<br>H -5.5467737111 3.5736006841 9.0424788609<br>H -6.3057179792 3.6284680812 7.7257252698<br>O -5.8811412933 -1.8625283442 -7.6769763926<br>H -6.0592414181 -0.972874107 -7.3057501551<br>H -6.6271987916 -2.3982115574 -7.3235041353<br>O -9.1379948569 3.8465104426 -2.9728879569<br>H -9.5357726942 4.6650602417 -2.6046546471<br>H -9.0630122835 4.0621411324 -3.9267797366<br>O 8.1540995044 6.3243536969 -2.5019028755<br>H 7.188194551 6.4308519496 -2.6484683588<br>H 8.4304399594 5.8811679059 -3.3283542343<br>O -5.5481042361 9.7244150516 -7.1599594712<br>H -4.8311846362 9.9107485784 -7.801589109<br>H -5.0452634608 9.5222820306 -6.3336919929<br>O 1.5575786576 12.3629981084 2.5618650318<br>H 1.9793234285 12.9284427353 1.8944854555<br>H 0.6172455211 12.3994270474 2.2864413055<br>O -5.3315341411 -2.5440555115 -10.2177543945<br>H -5.5248462038 -2.1729559076 -9.3287222418<br>H -5.0633098098 -3.4588752252 -10.0082227318<br>O 7.1358867496 -5.4682925648 -4.9997037641<br>H 7.7680962091 -6.1598591429 -4.7196513735<br>H 6.3831798621 -6.0008042432 -5.3324715068<br>O -6.785561003 0.1740170546 10.1922445992<br>H -6.771517513 0.1187954155 9.2260561239<br>H -7.2389818552 1.022073553 10.3555155456<br>O -1.6757378739 13.2947837961 -0.733252504<br>H -2.3229174923 13.3040474683 -1.4680968321<br>H -0.8254315622 13.3806833007 -1.1902556602<br>O -2.4162907509 0.3865192548 11.2364561621<br>H -3.2723844536 0.0540773619 11.5674030535<br>H -1.7665096331 -0.0876003413 11.7773986923<br>O -5.9208071339 -8.0807240889 4.4473893294<br>H -5.5608908564 -7.1659255806 4.5072426748<br>H -5.6363408299 -8.4657143777 5.2957526933<br>O -7.1614693447 3.4304351565 -9.4971954565<br>H -7.8602446615 3.2117080201 -8.8384425269<br>H -7.2865611997 2.7371305391 -10.1722310197<br>O -11.7222780334 3.6685478146 0.5084725403<br>H -12.19175437 3.5337038623 -0.3384780184	

H -11.5635843519 4.6366127966 0.5016424078<br>O 8.1244199527 -2.2199829114 -7.3648317262<br>H 7.783076548 -1.5427310804 -7.9909559489<br>H 7.7783918817 -1.8900757545 -6.5066099898<br>O -5.4084835269 -10.3170449108 -1.6477478795<br>H -5.960036897 -10.6548132991 -0.9147886219<br>H -4.8165561034 -9.694583376 -1.1796407575<br>O -1.2649571752 -6.6238730228 -10.8402859804<br>H -0.4219436215 -6.1390946915 -10.948378257<br>H -1.8541384716 -6.1359463443 -11.4663383726<br>O 11.0684396875 6.2746101886 -4.5796582508<br>H 11.5085052064 5.6330654757 -5.1890133736<br>H 11.3355314788 5.9433489735 -3.6922515334<br>O -6.2584922039 8.5096038867 -0.6480865127<br>H -6.2684704414 9.1754972924 0.0690679616<br>H -5.6734377686 7.8161967729 -0.2682816744<br>O -7.7987194325 11.3951395232 -4.0059819703<br>H -7.3121469958 11.8785357317 -3.3256586127<br>H -7.4415176078 11.7515704386 -4.8379973125<br>O 2.4052803695 8.4716671097 -5.5957147508<br>H 1.6211484923 8.1935164564 -5.0729189707<br>H 2.8084418459 9.1309388308 -4.9999119535<br>O -3.0076562546 -14.164514153 -0.9729974627<br>H -2.1641388867 -14.4059091507 -1.3828619741<br>H -3.3840310165 -13.5545864574 -1.6334695741<br>O -1.0677320597 -1.1803410771 -12.2261684191<br>H -0.8036526448 -1.6875806993 -11.431134032<br>H -1.2550024723 -0.2870864027 -11.8622835824<br>O 2.4577783558 -4.6081318715 8.7828991942<br>H 1.5992418808 -5.0810484308 8.8529089823<br>H 2.2088500279 -3.8289235117 8.2343516178<br>O -1.2635746648 1.3801387328 -11.3355343917<br>H -0.9495690507 2.0998389654 -11.9177744376<br>H -2.0140524876 1.790689951 -10.880662221<br>O 3.4013642043 11.4079518632 6.7241809668<br>H 4.3292658166 11.21005643 6.9215021423<br>H 3.4719004174 11.7520175823 5.8040887815<br>O 14.2197946252 0.0496944494 -3.2769864597<br>H 15.08778502 0.4211705702 -3.4532337775<br>H 13.6372747461 0.8367904647 -3.262403764<br>O -4.4839868112 -7.0612566513 -8.0454708491<br>H -4.7769449518 -7.9788588893 -7.9214264923<br>H -3.5017477238 -7.142912425 -8.0402604468<br>O -5.5972821882 2.3692549287 12.3144984005<br>H -5.2101064799 3.2356220933 12.5361336351<br>H -6.4705957438 2.5966141421 11.957607534<br>O 2.5564349529 13.9780005075 0.4951042772<br>H 2.4920167851 14.9356831816 0.4550514601<br>H 2.0630334739 13.6921382452 -0.2975695515<br>O 8.9029957677 1.6017169611 -8.821975651<br>H 8.3447431601 2.1980840244 -8.27115288<br>H 8.3047255522 0.8310122815 -8.9517674041<br>O -0.5432911317 1.6337762445 13.8891794392<br>H -1.0459790321 2.2340748489 13.3112869434<br>H -0.0635568943 2.2349079738 14.4642490677<br>O 9.3261282103 -3.7740165112 -4.915544153<br>H 9.9402695488 -4.4455415683 -5.2485364119<br>H 8.4700431077 -4.2414703932 -4.9402916573<br>O -6.7773572431 12.0032760262 -6.4971501326<br>H -6.4051202917 11.1341905995 -6.7663830519<br>H -7.3948004306 12.2043410887 -7.2044056448<br>O -3.4190597435 -2.6762658124 -12.300587486<br>H -3.9211253562 -2.4023814711 -11.5098922027<br>H -2.6581393595 -2.0630323901 -12.3016857694	

O -11.9781613768 -5.0921736331 4.0827150526<br>H -12.7809609351 -4.8481900886 4.5890714766<br>H -12.0359021891 -4.4859112918 3.3116340317<br>O -8.8260586902 4.6592416375 -5.5134623395<br>H -8.1729088974 5.381234026 -5.3858238393<br>H -9.5427761537 5.1233060606 -5.9911875097<br>O -2.1324301118 -4.6324493234 9.7312476951<br>H -3.017431491 -4.871514961 10.0550066853<br>H -1.8653301992 -3.9106886406 10.3480884379<br>O -11.7358777604 1.9999519127 6.0300222455<br>H -12.306362689 1.5183053016 5.3858153104<br>H -11.7425770683 1.4019131298 6.8006627987<br>O -3.3677696794 10.6064142537 1.9823949704<br>H -2.5532993245 10.1289289441 2.2080551878<br>H -3.2754841912 11.4359716743 2.4931302713<br>O 2.3927215196 -6.342092769 -9.0426222985<br>H 2.4366842173 -7.3291921691 -8.9671628097<br>H 2.4176145807 -6.0655980862 -8.0995045126<br>O 2.4606691098 3.5374370897 -7.0794772909<br>H 3.3984337618 3.7860962767 -6.9853883021<br>H 2.0878144188 3.7901371723 -6.2073620821<br>O 6.2627178146 -8.6035540274 1.7368505006<br>H 5.8170043496 -8.646026438 2.6123456382<br>H 6.3673999142 -9.5498293918 1.5153081068<br>O 10.7704276013 1.1934552791 0.8586286357<br>H 9.8934331027 1.0986622386 1.2782237395<br>H 11.3706749326 1.2120829312 1.6219689985<br>O -8.4152334507 5.9106894018 -8.7827359345<br>H -7.9777911464 5.1723355732 -9.2328935615<br>H -7.6670552956 6.4079157905 -8.40740921<br>O -7.2128739553 1.1875085569 -11.1000953577<br>H -6.2934585037 0.9541709032 -10.8498955649<br>H -7.1453964411 1.2898588238 -12.0568220487<br>O -9.7233912444 5.6174052092 3.4644901349<br>H -10.4256584286 5.8393887339 4.1003803356<br>H -9.4056657775 6.5046544156 3.1835049123<br>O -9.673736651 -3.923901553 0.9243785091<br>H -9.3777760632 -4.7882918544 1.2945632656<br>H -10.5721088009 -3.8283966998 1.3081548527<br>O 6.4668755143 -0.6024056034 8.2597861009<br>H 7.2724217059 -0.7779976186 8.7820251252<br>H 6.487004819 -1.3110590251 7.581193121<br>O 5.5242906749 1.8478279103 7.8079439798<br>H 5.9111244215 0.9521627151 7.9272723972<br>H 4.5658126295 1.6544167994 7.8964073085<br>O 1.07927315 11.4664480792 -3.9892574749<br>H 1.97250649 11.0628336071 -3.9147678057<br>H 0.5381246497 10.9269773689 -3.3862928005<br>O -2.6012899587 11.2905854098 -4.7533225235<br>H -3.1418817331 10.4922702005 -4.9561256722<br>H -1.8696862779 11.2324808558 -5.4015946846<br>O 10.3325768377 -7.3757662641 0.3848775852<br>H 9.611835828 -7.2384345864 1.036368034<br>H 11.0116522349 -6.7545793272 0.7015025493<br>O -8.9950668707 -3.3206210241 7.139419235<br>H -8.4265608251 -3.8060996636 7.7674631229<br>H -9.2283750642 -4.0246175771 6.4978702381<br>O -12.0507958581 -7.3602435291 2.4601982088<br>H -12.1334082621 -6.9474902159 1.5836888579<br>H -12.1283325864 -6.6071168708 3.0703355223<br>O 2.7301955934 -0.6544020257 12.2184226683<br>H 3.4404016638 -0.8866629889 12.8374266668<br>H 3.1731300738 -0.7129826371 11.3590708156<br>O 12.531563444 -3.7869275214 3.7862039903	

H 12.5786952679 -2.8122327801 3.8803958603<br>H 13.1952417138 -4.0996571079 4.407836078<br>O 5.4660844471 -3.8662565693 -8.2923290557<br>H 5.2917051579 -3.655714318 -7.3516116279<br>H 6.3819244974 -4.2012722122 -8.2553798926<br>O -2.2846204335 -9.7657717834 -7.4725104073<br>H -1.542689397 -10.1877547856 -7.9190687221<br>H -2.1338846514 -8.8200894404 -7.6530998455<br>O 12.2452622777 -3.669457328 -0.3204136454<br>H 12.0833299581 -4.1096036956 -1.1732863096<br>H 12.6104784721 -2.8091462332 -0.5970269159<br>O -3.4288364812 -13.4477042454 1.5882976926<br>H -4.3596662404 -13.6472985536 1.7577526218<br>H -3.2913995814 -13.7934689736 0.675729809<br>O 5.0431387848 -6.8328182222 -6.0755524453<br>H 5.4657145743 -7.0803541379 -6.918506329<br>H 4.5400260532 -7.6381416342 -5.8276906262<br>O 2.6728764399 9.3979121848 8.4549382967<br>H 2.7580458501 10.1991417875 7.9025345731<br>H 3.2835948274 8.7875488721 7.9901617393<br>O 12.864777052 -1.0986616287 -1.0784071678<br>H 13.4152923009 -0.9255904584 -1.8606084214<br>H 13.2266732958 -0.4670664309 -0.430274532<br>O -6.8651173303 -10.889502949 0.6319783403<br>H -6.4226938446 -10.182512838 1.1501331994<br>H -6.7163257613 -11.6729053046 1.1962369159<br>O 7.9065546875 -5.0751990365 10.7882874662<br>H 8.591977691 -4.3730849323 10.6846991641<br>H 8.4067202944 -5.8182108949 11.1343816548<br>O 4.3297830169 7.9119903137 6.9776760937<br>H 3.7260130446 7.858798342 6.2060496691<br>H 4.8374951419 7.0663320243 6.8934993476<br>O 7.6965631156 10.6609755618 1.5086042915<br>H 7.446267481 10.7865833615 2.4462884644<br>H 7.3760592282 11.4693781469 1.0739823555<br>O -4.235041799 -2.2307842329 8.9058752191<br>H -4.4264229933 -3.0241700958 8.3877621221<br>H -4.2576038536 -2.548828407 9.8232352085<br>O -9.6204442412 -5.2720437281 5.3297660392<br>H -10.5141337879 -5.1933718542 4.9158045125<br>H -9.4188094335 -6.2179385231 5.2002772341<br>O 11.2447999914 3.1244901861 -8.7471450668<br>H 10.4087222995 2.6317129754 -8.7995643619<br>H 11.8027548399 2.6916680456 -9.3946083343<br>O -2.594226009 6.1246204842 -10.2444925526<br>H -3.3634614977 5.9003159284 -9.6896603391<br>H -2.62143444 5.4458536192 -10.9624690362<br>O -8.0702393155 4.3898013886 5.2885244817<br>H -8.7416345726 4.2515287569 5.986062033<br>H -8.6105262346 4.7294755204 4.5498183059<br>O 6.479451569 12.242135255 -0.5080352333<br>H 5.6135151484 12.124810623 -0.960370614<br>H 7.104149094 11.9353845056 -1.1869345061<br>O -1.3297724963 -2.5012178959 11.0892522034<br>H -0.847752458 -2.0250327376 11.8001602338<br>H -0.9600744452 -2.0667478764 10.2918075184<br>O 5.7273085981 5.7072814275 6.7948903711<br>H 5.7920824443 5.1995727768 7.6344046648<br>H 5.7670409322 5.0030275015 6.1210927351<br>O -0.8168657027 -10.6759341368 4.7659318233<br>H -0.8917691115 -11.454405978 4.1726999263<br>H -0.0131243891 -10.2129011249 4.4045969642<br>O -1.5693396306 -7.2795447212 7.3143048276<br>H -2.4802455166 -7.1278771382 7.6426792912	

H -1.4842635598 -8.261168497 7.3500857434<br>O -6.4291192365 -7.9759302866 -5.4422882235<br>H -5.6172987512 -8.2619218752 -4.9752969694<br>H -6.4396229778 -8.5650080662 -6.2214345882<br>O -2.8947472955 12.9050216558 3.3791138453<br>H -3.5401892278 13.6035744594 3.5205350127<br>H -2.4903797211 12.8010443773 4.2789777629<br>O 3.6919889444 0.4456676722 -11.4848307364<br>H 2.7816330817 0.528403821 -11.1211149408<br>H 3.7693479506 -0.510687678 -11.631488516<br>O 0.5373483839 11.3961574095 6.6991386281<br>H 0.6593125978 10.8649100638 5.888631793<br>H 1.4482590212 11.6714701185 6.8902825699<br>O 7.9877658692 2.7156700889 -4.5079851581<br>H 8.2011195945 3.6784157393 -4.5742794306<br>H 8.242620557 2.5036271557 -3.582090574<br>O 0.2774448493 -11.1946434194 -2.4364505341<br>H -0.5057594283 -11.1722672673 -1.8423111366<br>H 0.9712138241 -11.5896285618 -1.8705813891<br>O 6.4243188885 -10.8190159882 6.9014239652<br>H 5.5036435047 -11.0546474102 6.6905007467<br>H 6.9095316612 -11.5861109252 6.5504262394<br>O 3.4976478968 10.3042778167 -3.8808256038<br>H 3.9598972893 9.7012627222 -3.2492025088<br>H 4.1899019885 10.4430061185 -4.576322746<br>O -12.3032948035 8.8773201633 0.7895690646<br>H -13.1691647304 8.780437763 1.187475477<br>H -12.0276415249 7.9668372977 0.610328265<br>O 1.2574037811 10.4508394752 10.5118821008<br>H 1.9009933718 11.0174232279 10.9432737947<br>H 1.778070376 10.0205343439 9.8031414845<br>O 4.9420833126 -12.2754998302 3.4565400462<br>H 4.1103559799 -12.1169339615 2.9578388697<br>H 4.6707717261 -12.058828101 4.3639740732<br>O -4.9263748979 -0.3305581743 12.0364125393<br>H -5.6338576721 -0.2976143484 11.3514321435<br>H -5.0927218273 0.5018885623 12.5119080366<br>O -9.5356152083 9.6286561147 0.6693597245<br>H -10.4794472103 9.8530275231 0.7158321981<br>H -9.098228947 10.4931660092 0.497051404<br>O 6.9631354313 7.2861108102 -5.6576527849<br>H 7.2170414873 8.2210241259 -5.5338094941<br>H 6.1200277579 7.3504384105 -6.1608099177<br>O 3.031302922 -8.9107064405 5.8383018339<br>H 3.1048899569 -7.9344441859 5.9545039151<br>H 2.4075134862 -9.1435768024 6.5746449673<br>O -9.4555132715 -7.1617082357 -1.4612340804<br>H -9.3864864848 -7.9660566296 -0.9116535934<br>H -8.6192702902 -7.2009143044 -1.9814640038<br>O 10.4306900926 2.7694265306 4.0882746444<br>H 10.8215139546 3.4299965365 4.7020205866<br>H 9.7385385213 3.3027470046 3.6530432248<br>O -5.2248062576 0.9541659162 -13.3231131944<br>H -5.3806916558 0.0385113461 -13.6253501119<br>H -4.9137582723 0.8294215928 -12.4079702136<br>O -9.0976159641 -9.5991384639 -0.1611394241<br>H -9.1239768804 -9.9183906241 -1.0782981237<br>H -8.3262700912 -10.0920080809 0.1900259411<br>O -1.2870178883 -12.6899658029 3.0292109899<br>H -2.0628181277 -13.0279885206 2.5280260456<br>H -0.6618256326 -12.4788352112 2.3068994916<br>O 0.8422832729 -9.6958275866 -6.1853405172<br>H 0.1568025527 -9.0681627855 -5.8614722869<br>H 0.5436956854 -9.8930010863 -7.0895918902	

O -12.1474233304 -3.4514866951 1.9657891762<br>H -12.7368312828 -3.4589978459 1.1844793726<br>H -12.1471791973 -2.502540171 2.2070484993<br>O -8.775892081 2.8400622993 -7.4996293783<br>H -8.7299654784 3.439057265 -6.7244681967<br>H -8.8800764477 1.9589307558 -7.092139584<br>O -1.269809744 4.2980989278 -8.7028841321<br>H -1.6925055069 5.0431006172 -9.1738899983<br>H -0.3181995808 4.4727013971 -8.8859198827<br>O 5.0057160407 4.5193051683 -6.5559766038<br>H 4.8570012607 5.4920099709 -6.6047057647<br>H 4.847152617 4.3441346643 -5.605332599<br>O 4.3355693052 -8.5774774454 -1.866122773<br>H 5.138112733 -8.6568414929 -2.4298239735<br>H 3.7008311846 -8.1280742462 -2.4525548037<br>O 12.5101087077 1.3628539611 3.0465046736<br>H 13.2749222832 1.910434881 3.3075389287<br>H 11.7628100863 1.8650053939 3.4356537656<br>O 4.0715467111 11.7917557 -1.5658887318<br>H 3.2603704747 11.3905791598 -1.2000662589<br>H 3.9878349641 11.5820962045 -2.5069319145<br>O 10.6225486388 10.7424453387 -0.6428090448<br>H 10.7007211223 10.4805587535 0.2810187589<br>H 10.3698789428 9.9231627675 -1.0883622048<br>O 6.2455205102 0.4249626832 11.6409729882<br>H 6.036694531 0.0152317944 12.4961687609<br>H 5.5494470561 0.0553429388 11.0679179593<br>O -4.1104672493 -6.6422833744 8.0947209759<br>H -4.2753045673 -6.3779386448 9.0174589726<br>H -4.3348771865 -5.8137723739 7.6166538136<br>O -7.845851626 -3.5061854012 -6.8256595803<br>H -7.5010373506 -4.3851643866 -7.1017543905<br>H -8.2727858347 -3.7367888403 -5.9652960036<br>O 5.0577231016 2.4704236791 -10.3899654136<br>H 5.9716328522 2.4492422814 -10.7451821965<br>H 4.6640874032 1.6624147546 -10.7938561946<br>O 3.8761605778 -7.0345309421 8.8435201471<br>H 3.5588440245 -6.1377655841 9.0380163997<br>H 3.2432045854 -7.5882661986 9.3280667928<br>O 9.4551125732 8.5632022041 -2.0473170112<br>H 9.8547496502 8.6716596898 -2.9311519394<br>H 8.9225226846 7.7382874246 -2.1618388114<br>O -2.4115203586 4.2274416663 -12.0916317441<br>H -1.5379679063 4.0599490038 -12.4819049357<br>H -3.0229504302 3.9205890541 -12.7959145962<br>O 10.1313445031 0.3079642305 5.1857263902<br>H 10.5086028727 0.5529915736 6.0527295631<br>H 10.1031462552 1.1777617999 4.7329502278<br>O 7.2401803517 10.3432640121 4.2206936028<br>H 8.0527928754 9.8175549787 4.4009447239<br>H 6.9217734078 10.5512636686 5.1161622119<br>O 12.3782086997 -1.1007265127 4.2670393599<br>H 11.5001598339 -0.8041139022 4.5620848997<br>H 12.6429566352 -0.3594377663 3.6904909584<br>O 10.6995417123 5.5696647721 2.2129286365<br>H 10.5199932493 6.0599077223 1.3856919136<br>H 9.8231011707 5.19383276 2.4420468725<br>O 11.824993051 4.3747649686 -6.2757818519<br>H 11.7654884444 4.1760678312 -7.2245539766<br>H 11.4620196886 3.5607730331 -5.8780899867<br>O 7.8944515288 2.4768194536 6.7421884306<br>H 6.996911742 2.3030860426 7.1041144868<br>H 7.7178294268 2.4031626882 5.7829636064<br>O -7.2061291266 -5.0354138417 8.4467780083	

H -7.4853861158 -5.9139150673 8.1323433838<br>H -6.4305334413 -4.8360657962 7.886470258<br>O -5.9020001467 12.2161168847 -1.9576341338<br>H -5.5694707661 11.3366900359 -2.199513362<br>H -5.1202640049 12.7736575017 -2.0716628687<br>O 12.1289077177 3.0762977794 -0.6096294866<br>H 12.260609929 2.7283250871 -1.5122113447<br>H 11.5204112038 2.4186625851 -0.2113398202<br>O -9.6537759888 -8.5480181268 2.359871508<br>H -10.6068879801 -8.3114175509 2.4094839794<br>H -9.6040747601 -9.0585902209 1.5282533995<br>O -4.0206988436 3.2485847623 -14.0187556757<br>H -4.3900883543 3.4782363674 -14.8726537368<br>H -4.4774222566 2.4090709702 -13.7958987328<br>O -12.8870918444 4.2157722334 4.595041165<br>H -12.4869896871 5.0361718183 4.9399514755<br>H -12.5463037492 3.5425542627 5.2004960564<br>O -6.8912890042 -5.9706210017 -7.3238536555<br>H -5.9869073153 -6.2114259667 -7.6013785947<br>H -7.0216368278 -6.5549953498 -6.5559728519<br>O -4.4966603516 -11.1715836521 4.1130224838<br>H -3.9510948935 -10.3659003171 3.9871432466<br>H -3.8784910597 -11.7805090955 4.5639974808<br>O 1.0658404471 -5.2164608249 -11.0513739365<br>H 1.5286316461 -5.6205747558 -10.2811452499<br>H 1.7792484169 -5.1333814361 -11.7021974762<br>O -4.5863063799 -5.1282522002 -9.8868410182<br>H -4.58351227 -5.8657526208 -9.2406747703<br>H -5.1857920215 -5.4569035073 -10.5798124859<br>O 4.4970757273 7.1443315343 -6.6741457397<br>H 4.4126872549 7.3720088753 -7.6285373973<br>H 3.7076205762 7.5886384335 -6.2866011547<br>O 5.9199351427 -3.490491897 12.0789070842<br>H 6.6492076892 -4.0876068195 11.852441652<br>H 5.3798303806 -3.5114479619 11.2665025185<br>O -10.1273950702 3.9719001482 7.0607835203<br>H -10.0929730623 3.669147646 7.9839644189<br>H -10.7350808425 3.3271535921 6.6533359354<br>O 4.6724291012 -5.59555297 -10.1710225791<br>H 4.9332971259 -4.9139238767 -9.5170577907<br>H 3.832024764 -5.9307456679 -9.7885975275<br>O -5.5423963115 -8.6971859312 7.1282139837<br>H -6.4225670641 -8.3522977308 7.3660431503<br>H -4.9467598529 -8.007123476 7.4944141347<br>O -7.8216596652 2.7030004526 10.4715332059<br>H -7.3996479357 3.3047492429 9.8295933251<br>H -8.7546281728 2.7131685674 10.19358004<br>O 1.2717565471 -12.014279385 -4.8438000156<br>H 1.0964732297 -11.2250452445 -5.3899883901<br>H 0.9289948276 -11.7382351401 -3.9760181459<br>O -1.8664249664 12.5918415745 5.7725731729<br>H -1.0065033655 12.3206953686 6.1352040411<br>H -2.4915165628 12.311900812 6.4686960576<br>O 1.2752042259 0.9440459127 -10.5133197253<br>H 1.171069711 1.040168067 -9.5408840605<br>H 0.3469279308 0.9970436847 -10.8215288038<br>O 0.0915115987 -5.9172867877 8.9104385118<br>H -0.6491392686 -5.4203578618 9.3171585738<br>H -0.3851447381 -6.4137299836 8.2078238662<br>O -10.6755072187 -6.3871264086 -3.8602218078<br>H -11.6135212611 -6.307085506 -3.6242044088<br>H -10.3053244581 -6.8634496131 -3.1002687889<br>O -0.3021282258 10.9854956433 -6.2238525434<br>H 0.1899494274 11.2796458972 -5.4232092768	

H 0.4183753111 10.9203313669 -6.877536005<br>O 10.6740254715 -2.6724227589 7.8776065795<br>H 11.5160495957 -2.3007712106 7.5778645702<br>H 10.0747936176 -2.5185019822 7.1302877776<br>O -14.2263343143 -4.1574875971 5.3258990181<br>H -15.1395992048 -4.3577320178 5.5349565193<br>H -14.155370871 -3.201917285 5.5147988945<br>O 3.2061051626 -8.6641170994 -5.3174685588<br>H 2.9072294152 -8.1101580708 -4.5660783701<br>H 2.3601950268 -9.0395550044 -5.6391165002<br>O 1.016759749 13.0383440031 -1.6283768229<br>H 1.1569436895 12.1273843122 -1.3089467496<br>H 1.0617141119 12.9276128369 -2.5896931466<br>O 7.6523663836 2.410534096 -11.2101647888<br>H 8.2361010025 2.1727719794 -10.4729355015<br>H 8.2589215709 2.6329679919 -11.9188308483<br>O -6.2270169662 6.7275310318 8.9666973836<br>H -6.361145657 5.7692790416 8.8075287879<br>H -5.5964743285 6.7244665175 9.7061064318<br>O 7.3471085067 -12.6628648688 4.9960123864<br>H 6.5608469187 -12.8007044557 4.4500131584<br>H 7.9993923252 -12.3390055862 4.3576862928<br>O 9.121629891 -8.4604288948 -1.856724567<br>H 9.0099167389 -9.3320211029 -1.4223882314<br>H 9.6093442049 -7.9642779104 -1.1699789895<br>O -13.3232567348 -0.1748149411 -0.7060204967<br>H -12.5155543337 0.1663545875 -0.2646871723<br>H -13.9996241573 0.4590100311 -0.4179756339<br>O -0.037157022 -0.714524109 12.6023375232<br>H -0.1750802028 0.0649925333 13.1767485605<br>H 0.9327812991 -0.7100454838 12.4783219905<br>O -1.6686127532 8.9159272075 -7.2850209607<br>H -1.1829091766 9.6289352286 -6.821275186<br>H -2.377401933 9.4054221116 -7.7411864402<br>O 13.0069511125 4.2855903916 1.7128223257<br>H 12.801497685 3.9510765117 0.820791819<br>H 12.1920683303 4.7832554221 1.9357768423<br>O 5.8411862673 4.1961663267 9.0070327448<br>H 5.6988359865 3.3412502459 8.5423318266<br>H 6.4600252404 3.9248404697 9.7161326333<br>O 8.0558749548 -4.7874324553 -8.1421281421<br>H 8.1261087702 -3.8626869441 -7.8176830651<br>H 8.992725459 -5.0568910537 -8.1748279909<br>O 5.5398783202 -2.0588953142 -10.3406427978<br>H 4.7865631797 -2.2210354079 -10.9263216557<br>H 5.43758473 -2.7362692369 -9.6457371961<br>O -10.9918780337 1.9192586422 -2.9671490087<br>H -10.2400099076 2.5482387891 -2.886784057<br>H -11.69763938 2.3657402689 -2.4711870751<br>O 2.1199423179 -12.4233332035 -0.8653979101<br>H 2.4579187599 -13.2946910068 -1.0934440527<br>H 2.9550352412 -11.9031320828 -0.7280272748<br>O -8.0569113605 -10.0226760292 -2.7379766978<br>H -7.9053834197 -9.1072392888 -3.0073214721<br>H -7.1630736255 -10.3498940801 -2.5756765354<br>O -10.4262840118 2.5438496642 9.4637216215<br>H -11.1231550619 2.7102515852 10.1040892501<br>H -10.7063621191 1.7015302145 9.0469864279<br>O -14.8734047179 1.9685323331 0.231126498<br>H -15.7988482396 1.8686905259 0.4697698055<br>H -14.461931607 2.2733170325 1.0728212318<br>O 12.8903308058 -2.2323492862 -4.161518373<br>H 13.4878913284 -1.483270052 -4.0064401613<br>H 12.0778786311 -1.8075315807 -4.4768494702	

O -1.0145337355 -9.2931906135 -10.8982179106<br>H -1.0856539716 -8.3138982958 -10.8666805468<br>H -1.5774989368 -9.5253696258 -11.6390592568<br>O -10.5885352444 5.9559223355 -7.130870107<br>H -11.1597914313 5.2257436315 -7.4180070121<br>H -9.9304696789 6.0202687065 -7.855522103<br>O 8.6842202316 -11.0755932562 3.057938164<br>H 8.4452827174 -10.4050932111 3.7255807576<br>H 9.47831852 -10.6902382703 2.6370479799<br>O 10.765924179 -2.3950973741 -7.9106831332<br>H 9.8081740444 -2.409326991 -7.7232162464<br>H 10.9757060365 -3.3335092714 -8.020010154<br>O 2.6047203007 -8.9474316442 -8.7920440331<br>H 3.2671832526 -9.48885784 -8.3218955067<br>H 1.823435067 -9.521568379 -8.8018986529<br>O 10.7781569836 -5.2544471812 -7.933558697<br>H 11.3727271907 -5.812747658 -8.4400612141<br>H 10.9576391442 -5.5242193855 -7.0046083735<br>O 10.5993922249 7.264140851 0.0609561408<br>H 10.1649297131 7.6915725983 -0.6989815121<br>H 10.4910985131 7.9383932583 0.7561398557<br>O 9.6234815097 -3.1246990141 10.3364699745<br>H 10.109413016 -3.051282603 9.4917272976<br>H 9.2389504348 -2.2353344974 10.4283097521<br>O -7.2822330963 -3.0843010997 -11.9551068619<br>H -6.5576057132 -2.8206078946 -11.348222141<br>H -7.9974793166 -2.4782909647 -11.6746349222<br>O -6.1008412265 -12.6747593777 2.5733040666<br>H -5.5360465058 -12.1115145527 3.1465307815<br>H -6.68031379 -13.1108024134 3.2036701707<br>O -4.2449656377 4.7873578896 12.6028481121<br>H -4.1275440674 5.2369431175 13.4436097786<br>H -4.2827399331 5.5259247068 11.9618714091<br>O -0.7341665654 9.1759957357 12.1132009104<br>H -0.0392496729 9.7145890335 11.7092200827<br>H -0.2780783874 8.3340442441 12.2914110074<br>O -11.8920114071 -0.6647613555 -2.9916884408<br>H -11.4024660732 0.1768162802 -2.9705183444<br>H -12.5224630593 -0.5514957143 -2.2568353293<br>O -8.612683053 -7.8058743892 4.7947622942<br>H -7.6721491475 -7.9574931247 4.5683008898<br>H -9.0690791841 -8.1996111154 4.0226585741<br>O -4.0969970471 6.7726314398 10.7448027883<br>H -3.5967001646 6.1523985754 10.1755838848<br>H -3.6078907088 7.6130092079 10.6290703724<br>O 6.0135622305 8.5159087363 9.2631290075<br>H 5.9533496004 7.8941870193 9.9893791504<br>H 5.3641738136 8.195283435 8.626140638<br>O -6.2564241762 -9.2828334393 -7.8579032247<br>H -6.3318321141 -10.1948098171 -8.149919651<br>H -6.7113165541 -8.7805912054 -8.5636313293<br>O -6.1600203157 7.6464335543 2.4264181213<br>H -6.0873096562 7.1008951612 3.2450364206<br>H -5.5594074449 7.1873233423 1.8059254738<br>O 6.4464705757 -7.0210693777 -8.4862295792<br>H 5.9331939567 -6.7174363386 -9.2530682907<br>H 7.0928121805 -6.2976735074 -8.3626737197<br>O -1.0149179485 10.7087361471 8.8756792886<br>H -0.3192303242 10.7096020285 9.5535744276<br>H -0.5151507779 10.9346391642 8.0678688314<br>O -8.8076112834 8.0162250376 2.7311059639<br>H -9.0834374924 8.6443485903 2.0317575608<br>H -7.8431950661 7.9332385496 2.570510551<br>O 1.1950812427 -1.5135475334 -13.5739955629	

H 0.9362599308 -1.8951735404 -14.4166602324<br>H 0.3302839582 -1.3893260884 -13.1163716779<br>O -2.3914666143 8.7669207668 -10.6420098623<br>H -2.4919349335 7.7914926373 -10.5870808919<br>H -1.4311235531 8.8800942011 -10.6048272788<br>O 9.0787052304 -7.3641856162 -4.3391309214<br>H 8.8446781737 -8.2067641106 -4.7791447283<br>H 9.1680698359 -7.6509648778 -3.4042898794<br>O 5.139364367 -1.3754481783 13.5243819244<br>H 5.2518998707 -1.6293625668 14.4430990593<br>H 5.4415437801 -2.1738940257 13.0350349274<br>O 3.9123956609 -11.4605721042 5.9423796949<br>H 3.591674194 -10.5427245867 5.8331834546<br>H 3.1512141509 -11.9146498335 6.3145985602<br>O -5.5988229216 -1.7262866724 -13.7754829537<br>H -4.7961302179 -2.1482060188 -13.4317163922<br>H -6.3099777828 -2.2309065972 -13.352410593<br>O -5.9776747321 10.1690746706 1.4832558324<br>H -6.0909220225 9.349539717 2.0052401025<br>H -5.0499104278 10.4107090742 1.6804637507<br>O 9.9733923301 9.2594232196 1.8596094026<br>H 9.1596630846 9.7646317914 1.6601544036<br>H 9.8659507656 9.0581760641 2.7990057023<br>O 0.2012928583 -10.4324010795 -8.8182979765<br>H -0.2175804522 -10.0008627115 -9.5995946069<br>H 0.2853570803 -11.3486286431 -9.097336322<br>O 1.3437805612 -9.521400454 7.7468316356<br>H 1.37586182 -9.1295084455 8.6339950013<br>H 0.3904907022 -9.7227462381 7.6328507444<br>O -1.2857335438 -9.9266352128 7.2491975492<br>H -1.118563758 -10.2702212136 6.3362036686<br>H -2.1444387365 -10.3518640543 7.4657566226<br>O 7.3293476394 3.0687109479 -7.2190055071<br>H 7.6255458139 2.9083686231 -6.3080098688<br>H 6.5658033598 3.6550861743 -7.0880708623<br>O 12.1365275225 -7.9598002783 3.1715319596<br>H 11.5147545904 -7.7581122828 3.8843864777<br>H 12.1857101806 -7.132218697 2.6689570031<br>O 11.7314709424 -4.3690928368 -3.0097197855<br>H 12.2600227785 -3.6672997704 -3.4439107755<br>H 10.824080232 -4.1132369875 -3.2093703676<br>O 8.5750082744 5.2616891581 -4.9237141897<br>H 8.0379305828 5.9524103414 -5.3740823592<br>H 9.4843436665 5.6194249248 -4.9972466202<br>O 11.3510451072 4.6996694652 5.7280480682<br>H 11.457011434 5.6034369928 5.3755535971<br>H 10.6541964566 4.8105420662 6.4055989533<br>O 14.4556898009 3.2155144267 3.6272466409<br>H 15.3652410955 3.4964646698 3.5018882916<br>H 13.9723352953 3.7069084374 2.9172868953<br>O 10.387711738 8.7985760214 -4.6325881987<br>H 10.6498948129 7.8436120284 -4.6767021781<br>H 11.1603117072 9.2445048965 -4.9903775068<br>O -13.2359743823 3.1002623972 -1.6808988587<br>H -13.9029427728 2.6542602395 -1.1227715317<br>H -13.7668927225 3.5506212026 -2.3441278783<br>O 8.268276972 11.0584899937 -2.3414154508<br>H 8.4573926227 10.1343633198 -2.1039391983<br>H 9.0542386906 11.5163404072 -2.0144424115<br>O 1.2904624563 4.7970091567 -9.1875261977<br>H 1.73024956 4.3309378193 -9.9320203387<br>H 1.7427159624 4.416392524 -8.4092535643<br>O 1.9546980485 10.1898078699 -7.6743988079<br>H 2.8535221303 10.3904805831 -7.9803064736	

H 2.1121460521 9.5233215777 -6.9776837532<br>O 6.5169216107 -11.2988251081 1.450632127<br>H 7.3394259849 -11.3284734956 1.9795703812<br>H 5.8886760333 -11.7536249663 2.0492048901<br>O -11.2809916518 6.1478783735 5.7031535817<br>H -10.8739086209 5.5681067492 6.3674182492<br>H -10.7303927525 6.9573886426 5.7593172078<br>O 0.148190017 3.2458521593 -12.7970396514<br>H 0.8702084498 3.6716293633 -12.3107530219<br>H 0.6301471848 2.6238344576 -13.370149059<br>O -3.497304831 10.3220014838 -8.8180513165<br>H -3.4654451284 11.2020292064 -9.2038538731<br>H -3.1898637842 9.7496490599 -9.5630156<br>O -4.065025531 -2.8623200747 11.6132190835<br>H -4.4327188763 -2.0144458811 11.938205911<br>H -3.1060548078 -2.7092618237 11.6527935699<br>O -2.7447417456 9.0802248868 10.2969536704<br>H -2.2109012287 9.596158122 9.6652391088<br>H -2.1859448434 9.1264606985 11.0992677146<br>O 13.8824544932 0.9286733441 0.5212251726<br>H 13.6885065682 1.7928830887 0.1418465225<br>H 13.5609766555 1.0042238675 1.427749479<br>O -6.2252124101 6.3225131331 4.7258696878<br>H -6.8057429303 5.5743729736 4.9582521006<br>H -6.3997760894 6.9585780068 5.4429678884<br>O -4.5582008323 13.4367413673 -5.457390257<br>H -5.3755447358 13.0783900646 -5.8331481954<br>H -3.9458933087 12.6942980415 -5.493765645<br>O 9.3099730289 8.6624647851 4.6308799774<br>H 8.8231010939 8.0350438468 5.2145872586<br>H 10.1971236506 8.2711079434 4.6245594958<br>O 3.5286672413 -4.9220329108 -12.4934505473<br>H 3.9078127321 -5.4358855404 -13.2109425784<br>H 4.0452432103 -5.2170520952 -11.7114740051<br>O -9.3223680205 -4.3033915806 -1.6964742521<br>H -9.4171821157 -5.2693653717 -1.6878797365<br>H -9.4908047164 -4.0708140723 -0.7562073596<br>O 8.4176499596 -0.6431110523 10.2262770717<br>H 7.7879042328 -0.2746242629 10.8687147329<br>H 8.8556196688 0.1478127916 9.863290606<br>O -0.6844445704 -14.0165771563 -2.6954372332<br>H -0.4315731325 -13.0922808357 -2.7968671238<br>H -0.5639968642 -14.3798914269 -3.5748683123<br>O 5.9867119581 10.1330276261 6.7888596308<br>H 6.4539811514 10.0092599342 7.623788704<br>H 5.4258192382 9.3381998023 6.7425960849<br>O 4.3482564755 -11.0472102767 -0.6034747292<br>H 5.1460675049 -11.077848942 -0.0559883858<br>H 4.3989303621 -10.1713440092 -1.0316047081<br>O 5.2868961388 10.4219939541 -5.7957179505<br>H 5.1867350413 10.398993135 -6.7647930453<br>H 6.2457387799 10.3227243803 -5.6627385405<br>O -6.4639874446 -11.2580628613 6.0439589219<br>H -5.8579774474 -11.2139313034 5.2878908172<br>H -6.3810985589 -10.3895125898 6.4488342297<br>O 11.0837706726 -5.9120220576 -5.3649497123<br>H 11.6090279901 -5.6944611971 -4.57967185<br>H 10.4000796094 -6.5177457041 -5.0038785495<br>O -9.4911599893 -1.8026187835 -8.0301197128<br>H -8.8759585354 -2.4545393891 -7.6223502494<br>H -10.2703624438 -2.3317961649 -8.2192395596<br>O -4.8975220556 -5.1413099772 10.2896330313<br>H -5.820928104 -5.0605189511 10.0030908055<br>H -4.8158017109 -4.4482475425 10.9686097365	

O 9.2462296112 4.6650186523 7.471730826<br>H 9.270990721 4.5636550598 8.4281334838<br>H 8.7410253965 3.8721254685 7.1804554003<br>O -8.7611542196 -1.1524430704 -10.7261092007<br>H -9.0253975046 -1.2151049098 -9.7969895664<br>H -8.3517358783 -0.2742149614 -10.7870012116<br>O 10.6693382612 -9.8081241027 1.6882071363<br>H 11.3655670733 -9.4619172558 2.2733385529<br>H 10.5563176816 -9.0694534184 1.0651961572<br>O -7.8345996951 11.6444675816 0.0903815779<br>H -7.3123492084 12.0138785679 -0.639267829<br>H -7.1506990656 11.2647955673 0.6718581756<br>O 13.0667050405 -1.4538242478 6.9201249949<br>H 14.014067128 -1.5452115265 7.0432005724<br>H 12.9671562274 -1.3310924343 5.9609912499<br>O 8.1471940772 6.8755168445 6.2159046785<br>H 7.2185882478 6.621763658 6.3582986049<br>H 8.6245011475 6.1763658394 6.6934954315<br>O 9.3040548294 1.5880707344 8.8831019627<br>H 10.1886440565 1.4046962133 8.517851993<br>H 8.8027193091 1.8515849111 8.0858978045<br>O -8.0324568273 -7.514948976 7.4237826208<br>H -8.794642519 -7.8514825274 7.9040615981<br>H -8.30568071 -7.6117814976 6.4858897512<br>O 1.4939381873 -7.9375125664 10.0728222695<br>H 1.1895969901 -8.0002712853 10.9812455651<br>H 0.9765077693 -7.1901191905 9.7116406232<br>O 7.4543642681 2.7892235163 10.6714393467<br>H 7.0515438916 2.0373890184 11.1376203105<br>H 8.207542904 2.3896779928 10.2107402079<br>O 5.1662111477 5.0123609763 -9.3508151617<br>H 5.1491875756 4.15916536 -9.8198367188<br>H 5.2192437753 4.7476631447 -8.4231664699<br>O -2.8560868734 -13.0487816868 5.2748352302<br>H -2.2155511932 -13.0497326858 4.5421954257<br>H -2.4189317214 -13.5625740419 5.9559516792<br>O -11.7661774163 -6.3440943022 -0.1335854262<br>H -12.3702857402 -6.1826481301 -0.8709454894<br>H -10.9500618204 -6.6250931827 -0.576561883<br>O -7.36353811 -7.5243456634 -9.6562368676<br>H -7.2069884469 -6.9662491442 -10.434412295<br>H -7.4968209851 -6.8776624425 -8.9478772452<br>O 2.4673092156 3.3515404706 -11.083262058<br>H 2.0979462597 2.455598265 -10.9771330505<br>H 3.4219753509 3.2052105863 -10.9715049516<br>O 7.8564914328 9.8282108054 -4.932545383<br>H 8.7811648726 9.512926059 -4.937148461<br>H 7.838566284 10.3989518474 -4.1460164745<br>O -6.8388523228 8.1212718413 6.7281691094<br>H -6.7183948091 7.5843926639 7.5390325834<br>H -6.3963585913 8.9468042635 6.9591818616<br>O 1.8918764603 1.2501626306 -13.7449941485<br>H 2.71754308 1.2427076484 -13.2483441622<br>H 1.6516885775 0.3138612877 -13.7868408988<br>O -11.5320718771 1.0409480596 -5.4897134097<br>H -11.3331521353 1.4622021948 -4.6311492149<br>H -12.0781114741 0.2898560117 -5.2314924413<br>O 11.6125652774 7.0477809336 4.2635891933<br>H 12.4049444074 7.4806837331 3.9360325612<br>H 11.2996763829 6.5343842254 3.4876442202<br>O 4.4055266169 7.6222475142 -9.2984788798<br>H 4.818702507 6.753605389 -9.4928954458<br>H 3.5387661934 7.5292616734 -9.7648812952<br>O -13.486296989 -2.9270942234 -0.3557138914	

H -12.9581827876 -3.1654759566 -1.13251853
H -13.5809713516 -1.9641170317 -0.4459616475
O -9.0484704016 0.3845840944 -6.4719797912
H -9.9209017822 0.5381218667 -6.0632948559
H -9.2190310368 -0.3926213504 -7.0421192271
O 11.5828441695 0.9938224284 7.4248665245
H 12.2210082322 1.6290941304 7.0349350005
H 12.1293953823 0.2132412152 7.5879185089
O 3.532398012 -12.769320442 -6.1398228767
H 3.7249126573 -13.665258986 -5.8601265404
H 2.7128703816 -12.5528923455 -5.6514652748
O 6.6564583269 -8.8278084254 -3.1552844725
H 7.4427790563 -8.6393839171 -2.6167571135
H 7.0326267997 -9.2590076853 -3.9367529993
O -13.0966948356 -5.7257209448 -2.6110553716
H -12.714583905 -4.8244231718 -2.649605471
H -14.0430476204 -5.5665757848 -2.6173047006
O -11.8464847612 -3.3442030332 -2.5778602489
H -10.9147786087 -3.5658149589 -2.4171172977
H -11.800794755 -2.414025568 -2.880034141
O -3.4772850572 11.5333361567 7.736475632
H -2.7331661252 11.2918717534 8.3065733943
H -4.1943564916 10.9610364214 8.047315418
O -9.3556730987 8.0355080369 5.5051794063
H -8.5024566424 8.1510444972 5.9574467612
H -9.1427853953 8.2288435259 4.5774340177
O -11.5906755051 3.2635511595 -7.4020263731
H -10.6983473918 3.0741662396 -7.7184078413
H -11.7787135609 2.521744315 -6.8115605107
O -6.6798716091 -5.6973533427 -11.7012944952
H -6.7584294465 -6.0486520581 -12.5909767115
H -6.9840901689 -4.76845681 -11.8057797545
O -3.8052519974 -10.8418681068 7.5498162213
H -4.2967530857 -11.6166709199 7.25888436
H -4.4543552034 -10.1239691932 7.4598465558
O 6.9009670357 -9.5656412169 -7.6048767715
H 6.7887200835 -8.7013165013 -8.0436032913
H 5.9964307387 -9.9269707297 -7.5993259264
O 8.7619379891 -10.6688882763 -0.3297676375
H 9.4302738088 -10.7048348358 0.3694493914
H 7.9756256163 -11.0443817928 0.0886593401
O 0.1446595308 9.0113785755 -9.4008349194
H 0.8116496468 9.5329916045 -8.9217581106
H -0.5010587295 8.8021305627 -8.707184148
O 4.2997393131 -10.3729960396 -7.1771823287
H 4.0083355567 -9.8509667947 -6.4073748919
H 4.1133601181 -11.2908556675 -6.8913516692
O -5.1226135189 9.4656487355 8.8597263394
H -5.6461767134 8.6774967464 9.0509861605
H -4.369888468 9.3807444515 9.4670700235
O 8.2511295557 -9.766892078 -5.3148703705
H 7.7850722309 -9.7037078293 -6.1782387846
H 8.6337327033 -10.6474414252 -5.3419198587
O 13.1803994588 2.7064168134 6.0788334475
H 12.6114369055 3.4979418964 6.0397949068
H 13.7925535216 2.8506234867 5.3395643629
O 1.9988200772 7.2117156209 -10.2960060549
H 1.6374855595 6.3748552535 -9.9533543045
H 1.2631786631 7.8349192271 -10.1648777018
O 4.7117199646 10.1517553007 -8.5039756295
H 4.6554844319 9.2271959738 -8.8359232817
H 5.0463663656 10.6329156287 -9.2649094287
